# Supplementary material for: Cu-Catalyzed Oxidative 3-Amination of Indoles via Formation of Indolyl(aryl)iodonium Imides Using o-Substituted (Diacetoxyiodo)arene as a High-Performance Hypervalent Iodine Compound
Source: Molecules. 2019 Mar 22;24(6):1147. doi: 10.3390/molecules24061147 (PMC6471596; doi:10.3390/molecules24061147)
Supplement: Supplementary file 1 [file molecules-24-01147-s001.pdf]

Supporting Information for

Cu-Catalyzed Oxidative 3-Amination of Indoles via Formation of Indolyl(aryl)iodonium Imides Using *o*-Substituted (Diacetoxyiodo)arene as a High-Performance Hypervalent Iodine Compound

Kazuhiro Watanabe and Katsuhiko Moriyama *

Department of Chemistry, Graduate School of Science, and Molecular Chirality Research Center, Chiba University, 1-33 Yayoi-cho, Inage-ku, Chiba 263-8522, Japan; cars9s66@chiba-u.jp

* Correspondence: moriyama@faculty.chiba-u.jp; Tel.: +81-43-290-3693

Table of contents

1. X-ray Diffraction Analysis of **2a**. - S2

2. ^{1}H and ^{13}C NMR Spectra of Materials. - S3

1. X-ray Diffraction Analysis of 2a.

Crystal data for 2a: Formula $C_{27}H_{28}N_2O_5S_2$, colorless, crystal dimensions 0.30 × 0.20 × 0.20 mm^3, Triclinic, space group P -1, a = 9.475(2) Å, b = 10.144(2) Å, c = 13.660(3) Å, α = 98.118(3) °, β = 91.348(3) °, γ = 100.871(3) °, V = 1274.7(5) $Å^3$, Z = 2, ρ_{calc} = 1.367 g cm^{-3}, F(000) = 552, μ(MoKα) = 0.250 mm^{-1}, T = 173 K. 7413 reflections collected, 5602 independent reflections with $I > 2\sigma(I)$ ($2\theta_{max}$ = 27.572 °), and 330 parameters were used for the solution of the structure. The non-hydrogen atoms were refined anisotropically. R_1 = 0.0508 and wR_2 = 0.1031. GOF = 1.015. Crystallographic data (excluding structure factors) for the structure reported in this paper have been deposited with the Cambridge Crystallographic Data Centre as supplementary publication no. CCDC-1860598. Copies of the data can be obtained free of charge on application to CCDC, 12 Union Road, Cambridge CB2 1EZ, UK [Fax: int. code + 44(1223)336-033; E-mail: deposit@ccdc.cam.ac.uk].

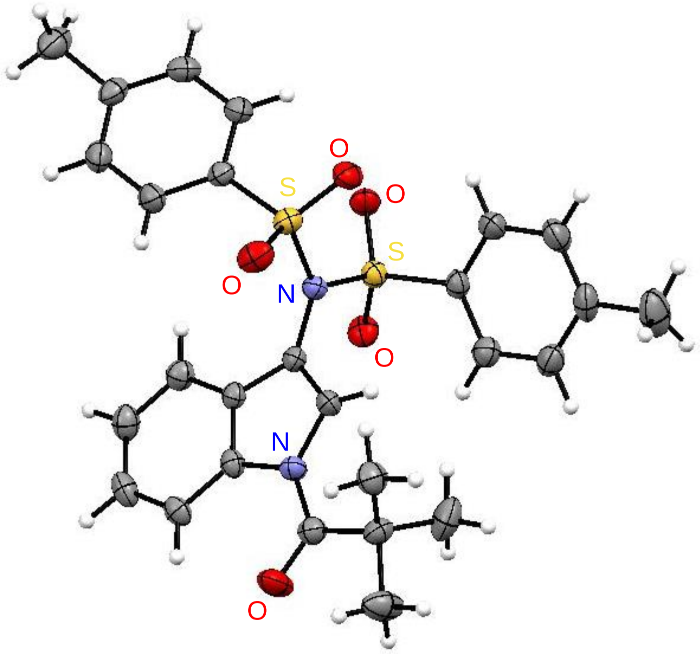

Figure S1. ORTEP drawing of **2a**. The ellipsoids correspond to 50% probability.

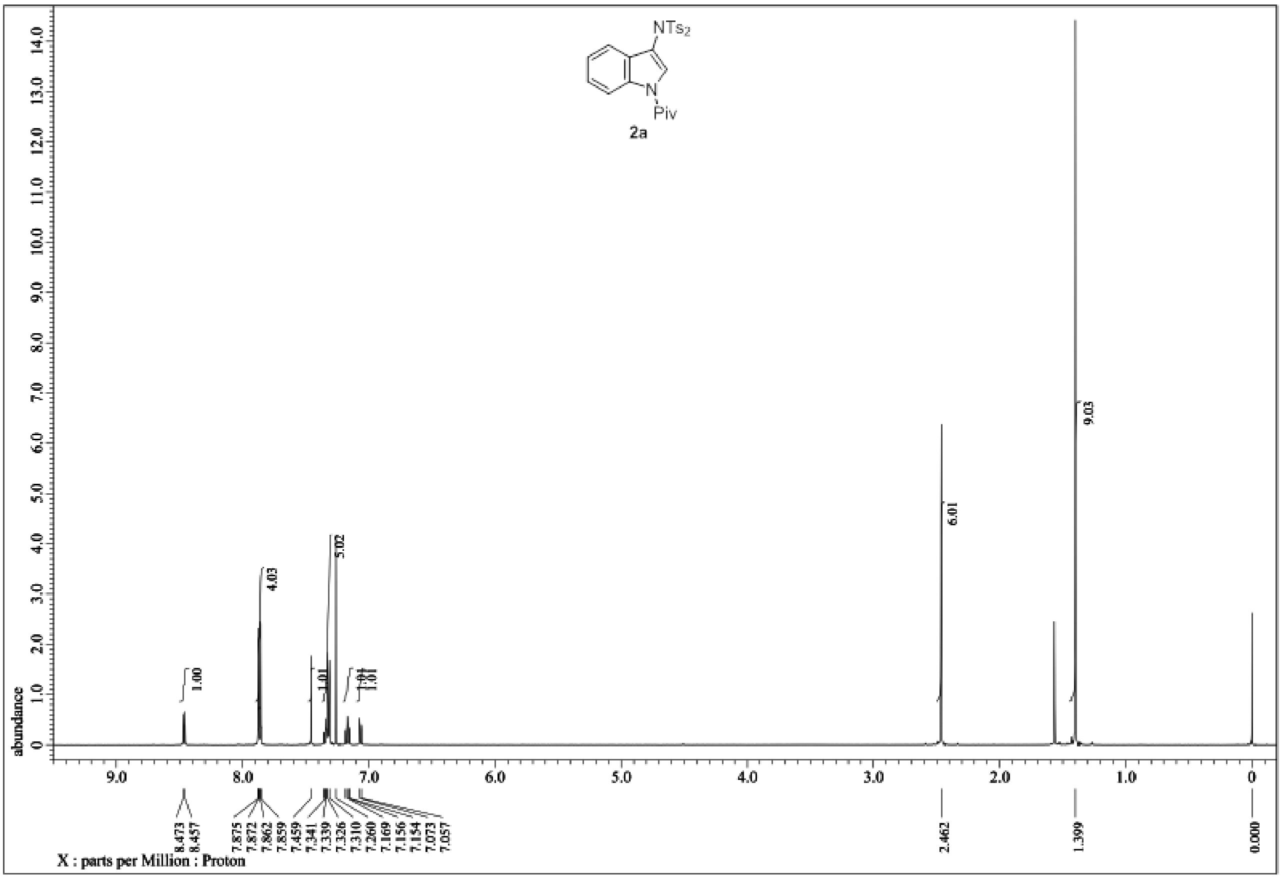

NTs2
N
Piv
2a
abundance
1.00
4.03
5.02
1.01
1.01
1.01
1.01
6.01
9.03
8.473
8.457
7.875
7.872
7.862
7.859
7.459
7.341
7.339
7.326
7.310
7.260
7.169
7.156
7.154
7.073
7.057
2.462
1.399
0.000
X : parts per Million : Proton

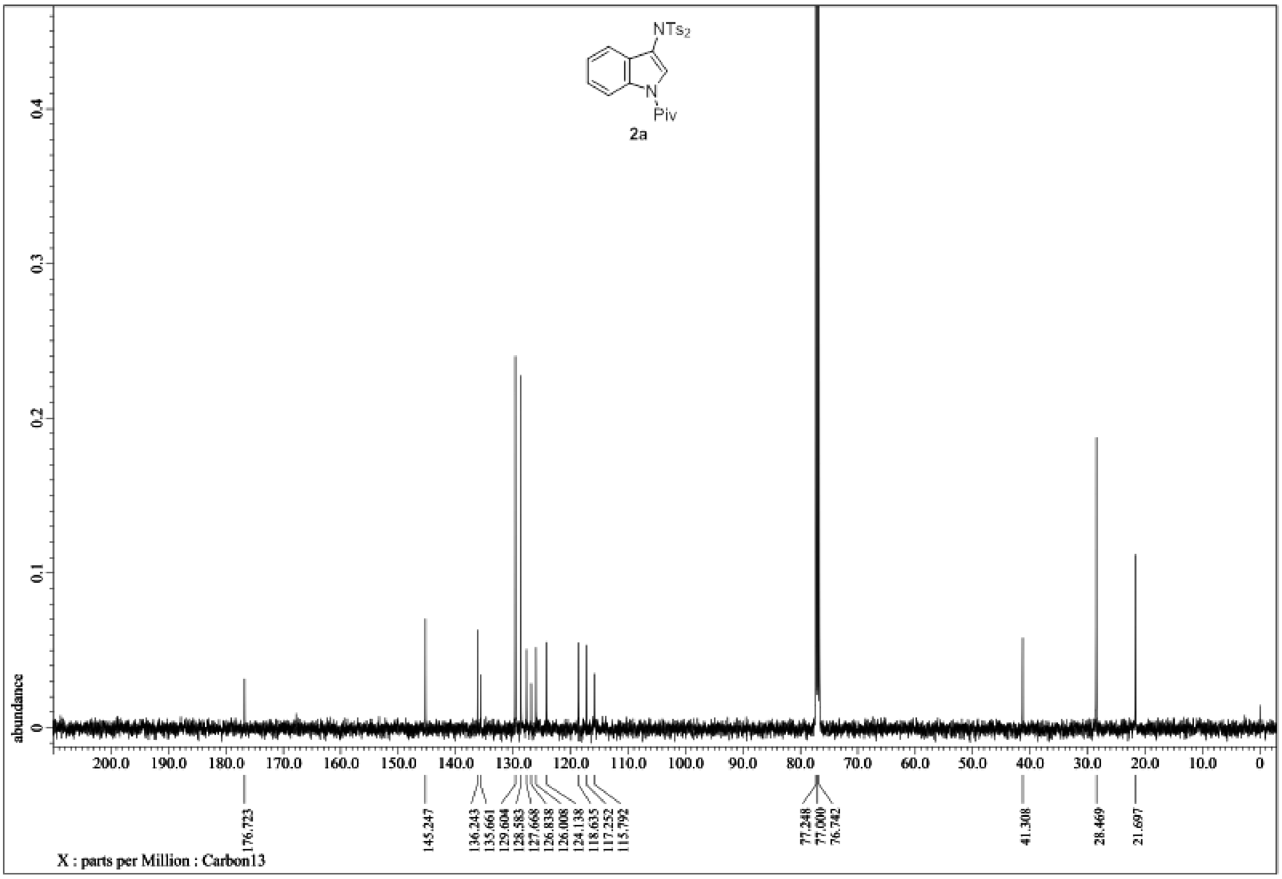

NTs2
N
Piv
2a
abundance
176.723
145.247
136.243
135.661
129.604
128.583
127.668
126.838
126.008
124.138
118.635
117.252
115.792
77.248
77.000
76.742
41.308
28.469
21.697
X : parts per Million : Carbon13

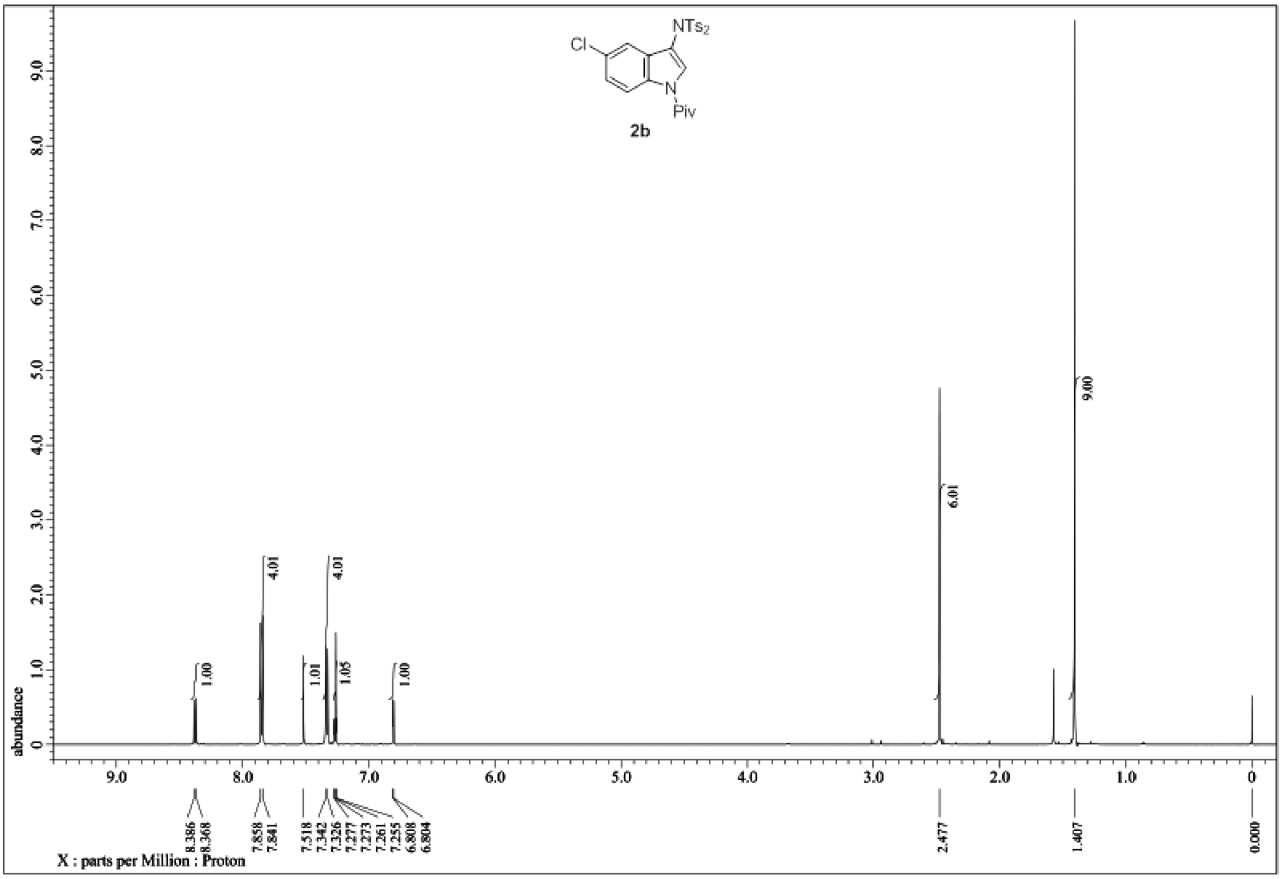
NTs2
Cl
N
Piv
2b
abundance
X : parts per Million : Proton
8.386
8.368
7.858
7.841
7.518
7.342
7.326
7.277
7.273
7.261
7.255
6.808
6.804
2.477
1.407
0.000
1.00
4.01
1.01
4.01
1.05
1.00
6.01
9.00

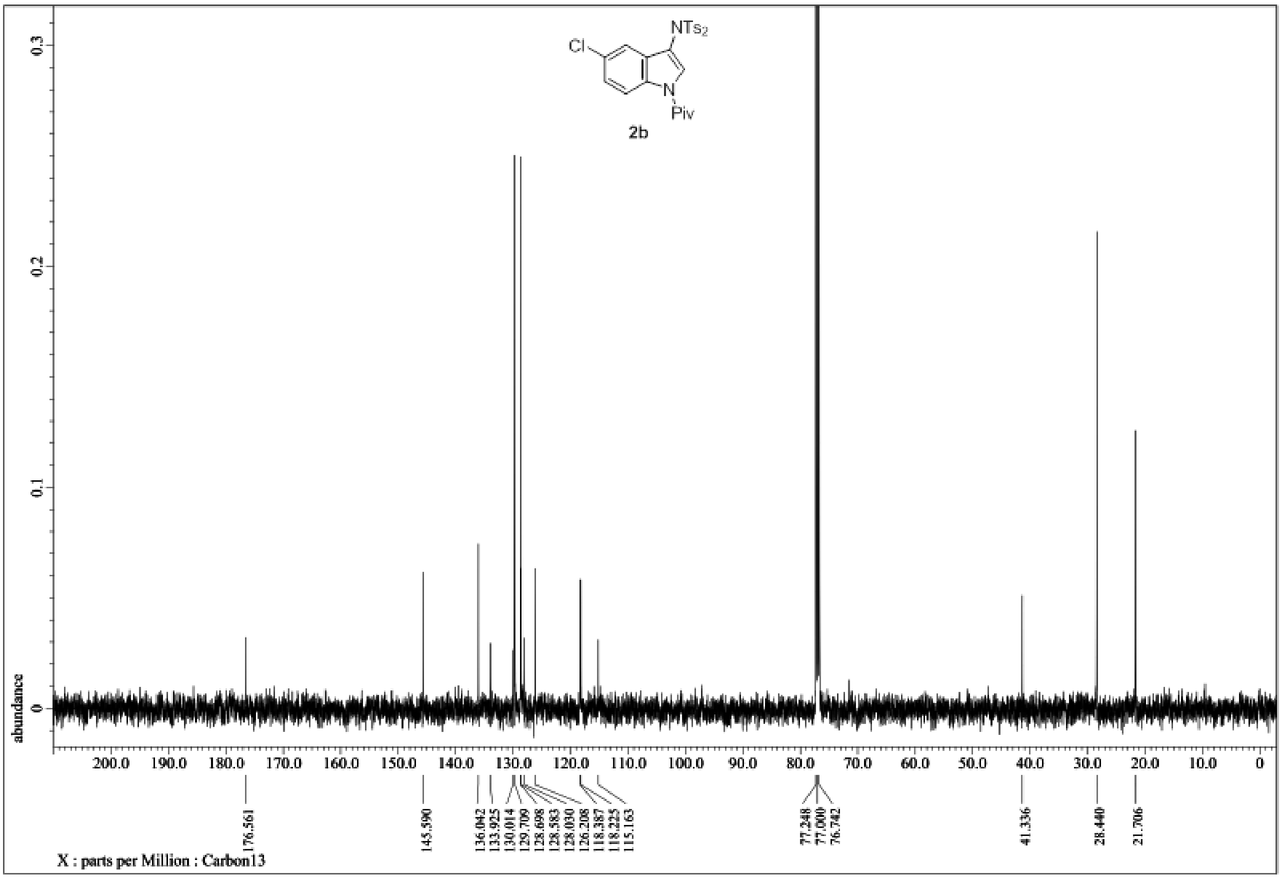
NTs2
Cl
N
Piv
2b
abundance
X : parts per Million : Carbon13
176.561
145.590
136.042
133.925
130.014
129.709
128.698
128.583
128.030
126.208
118.387
118.225
115.163
77.248
77.000
76.742
41.336
28.440
21.706

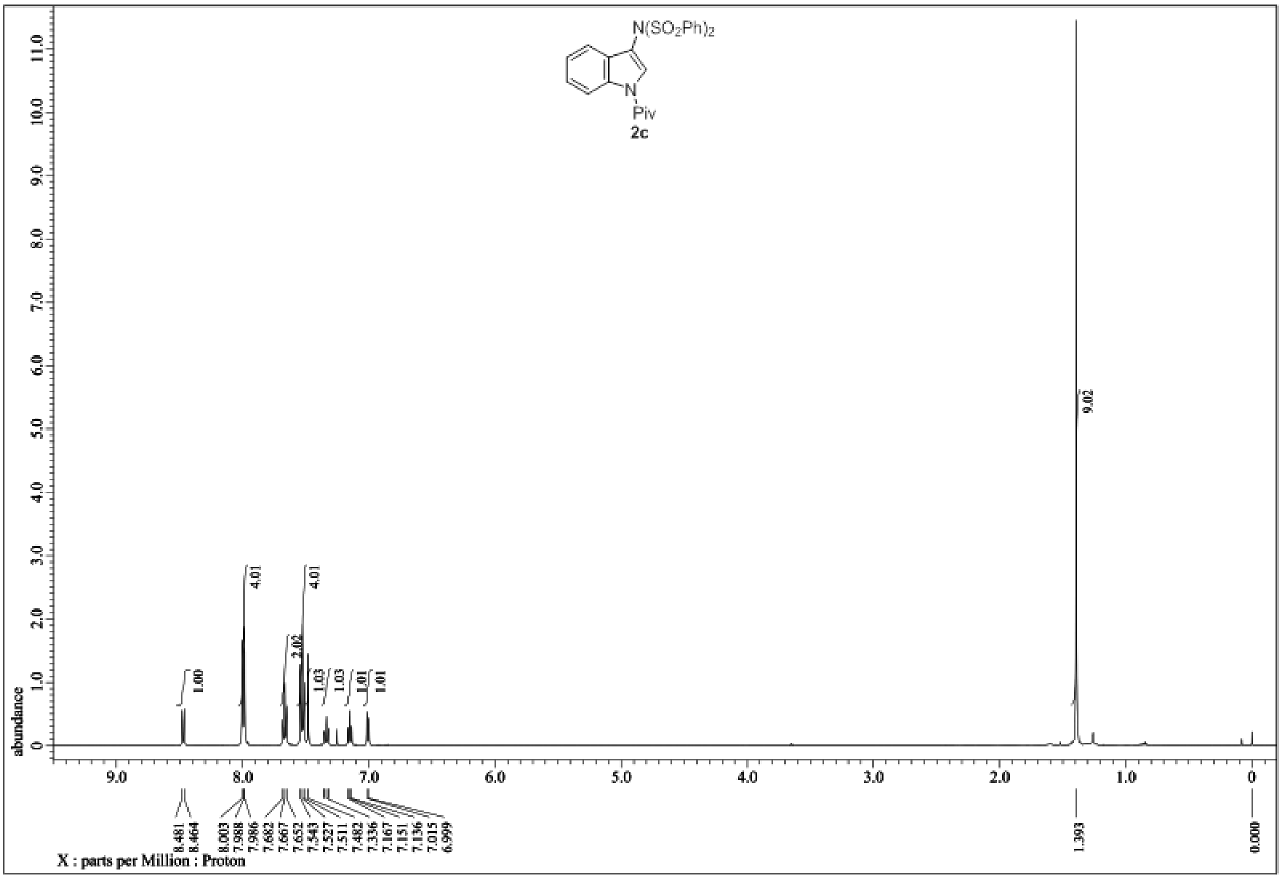
N(SO2Ph)2
N
Piv
2c
abundance
X : parts per Million : Proton
8.481
8.464
8.003
7.988
7.986
7.682
7.667
7.652
7.543
7.527
7.511
7.482
7.336
7.167
7.151
7.136
7.015
6.999
1.393
0.000
1.00
4.01
2.02
4.01
1.03
1.03
1.01
1.01
9.02

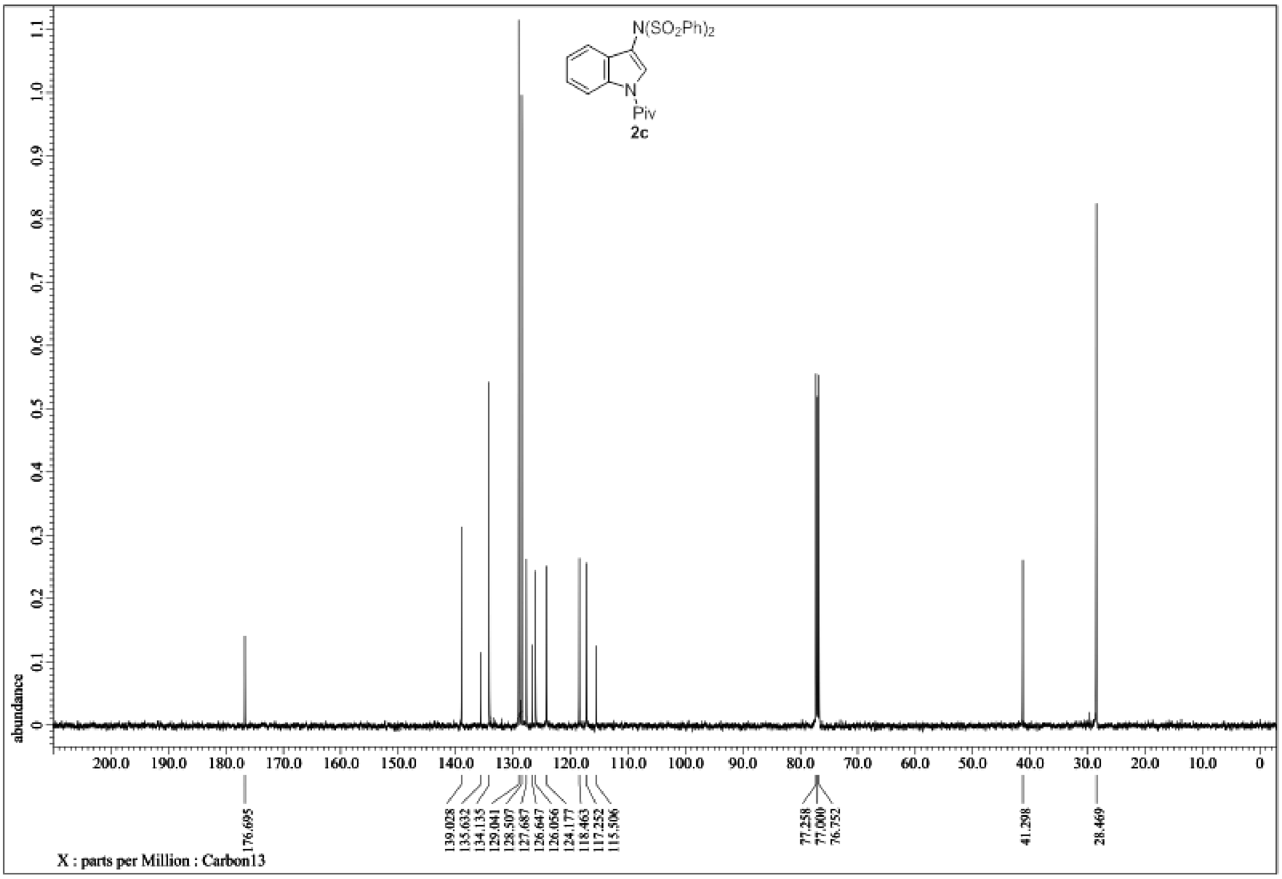
N(SO2Ph)2
N
Piv
2c
abundance
X : parts per Million : Carbon13
176.695
139.028
135.632
134.135
129.041
128.507
127.687
126.647
126.056
124.177
118.463
117.252
115.506
77.258
77.000
76.752
41.298
28.469

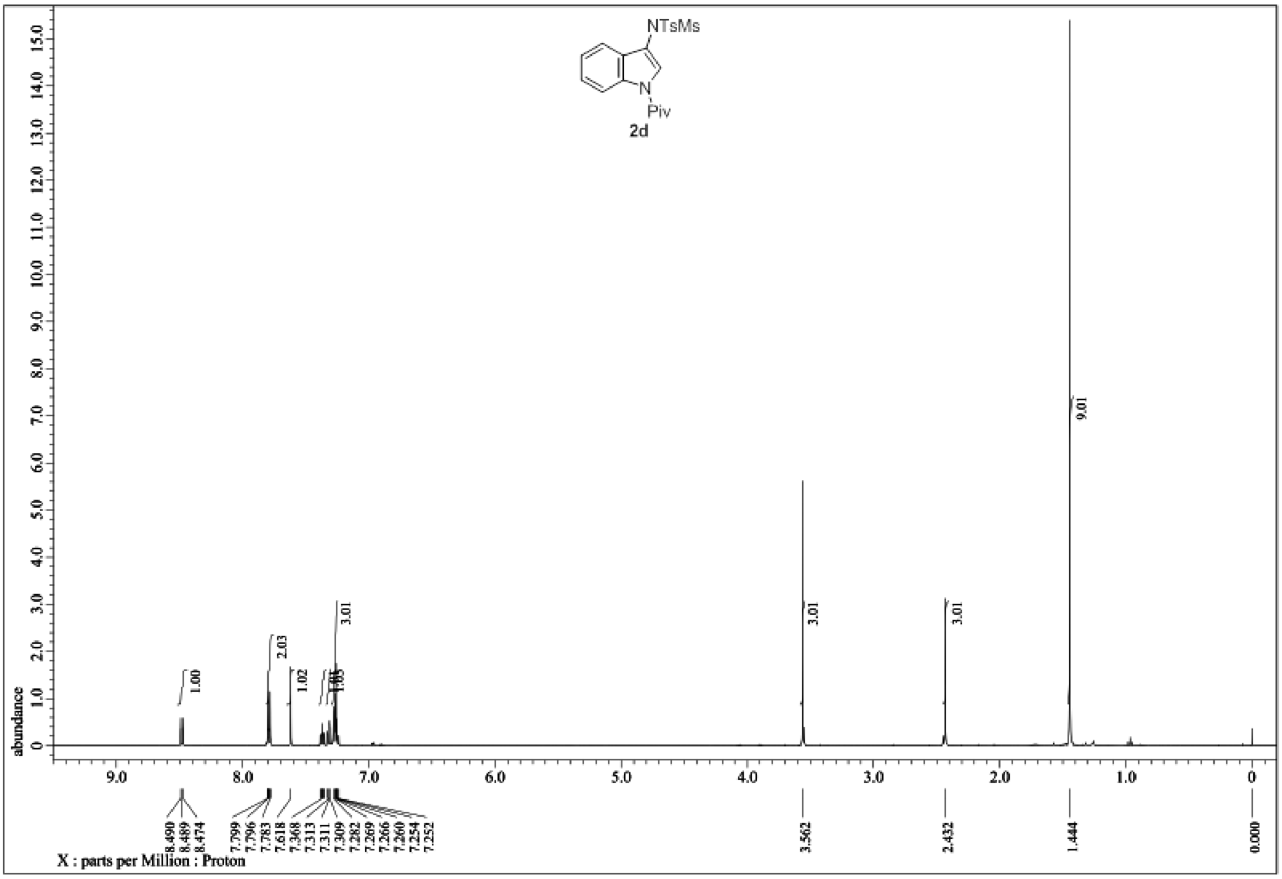
NTsMs
N
Piv
2d
abundance
X : parts per Million : Proton
1.00
2.03
1.02
3.01
3.01
3.01
9.01
8.490
8.489
8.474
7.799
7.796
7.783
7.618
7.368
7.313
7.311
7.309
7.282
7.269
7.266
7.260
7.254
7.252
3.562
2.432
1.444
0.000

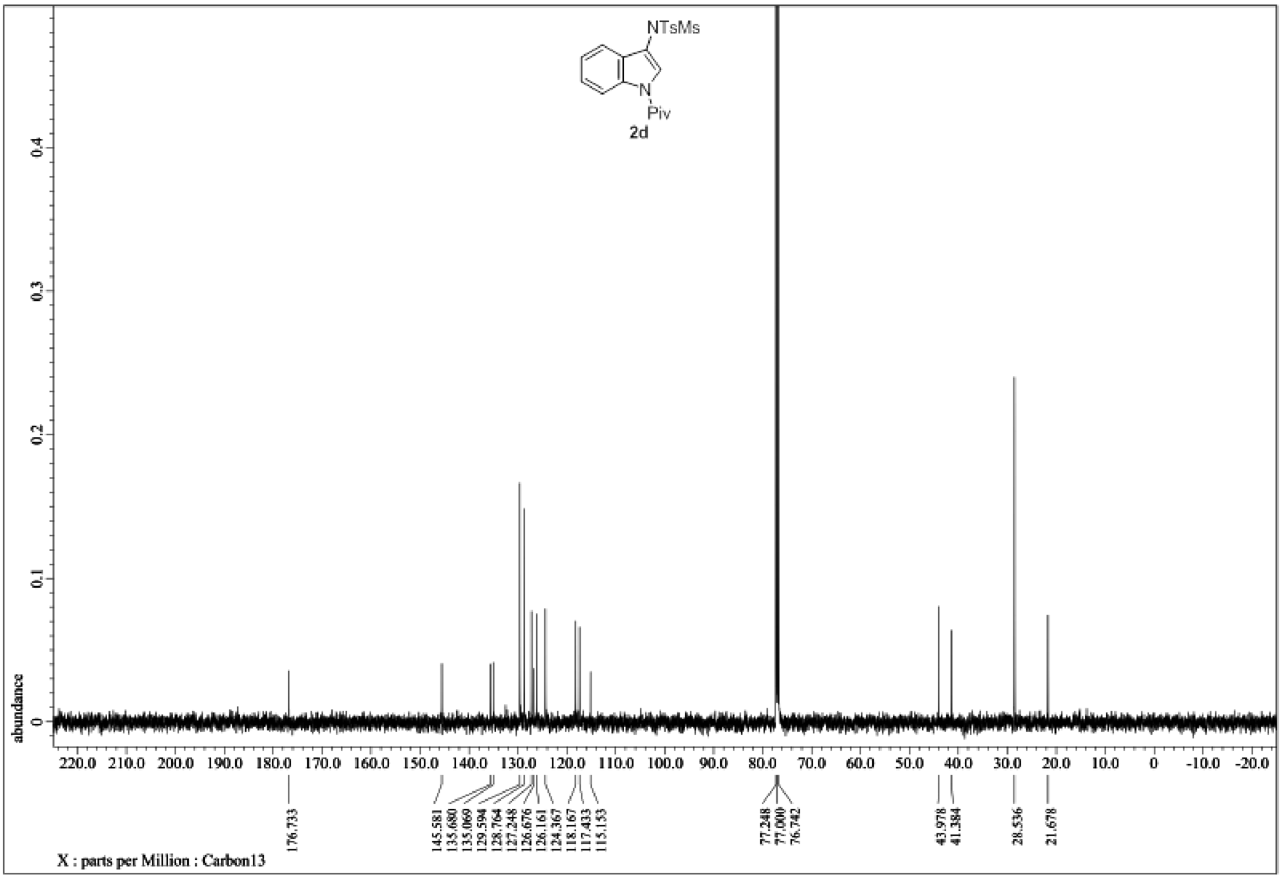
NTsMs
N
Piv
2d
abundance
X : parts per Million : Carbon13
176.733
145.581
135.680
135.069
129.594
128.764
127.248
126.676
126.161
124.367
118.167
117.433
115.153
77.248
77.000
76.742
43.978
41.384
28.536
21.678

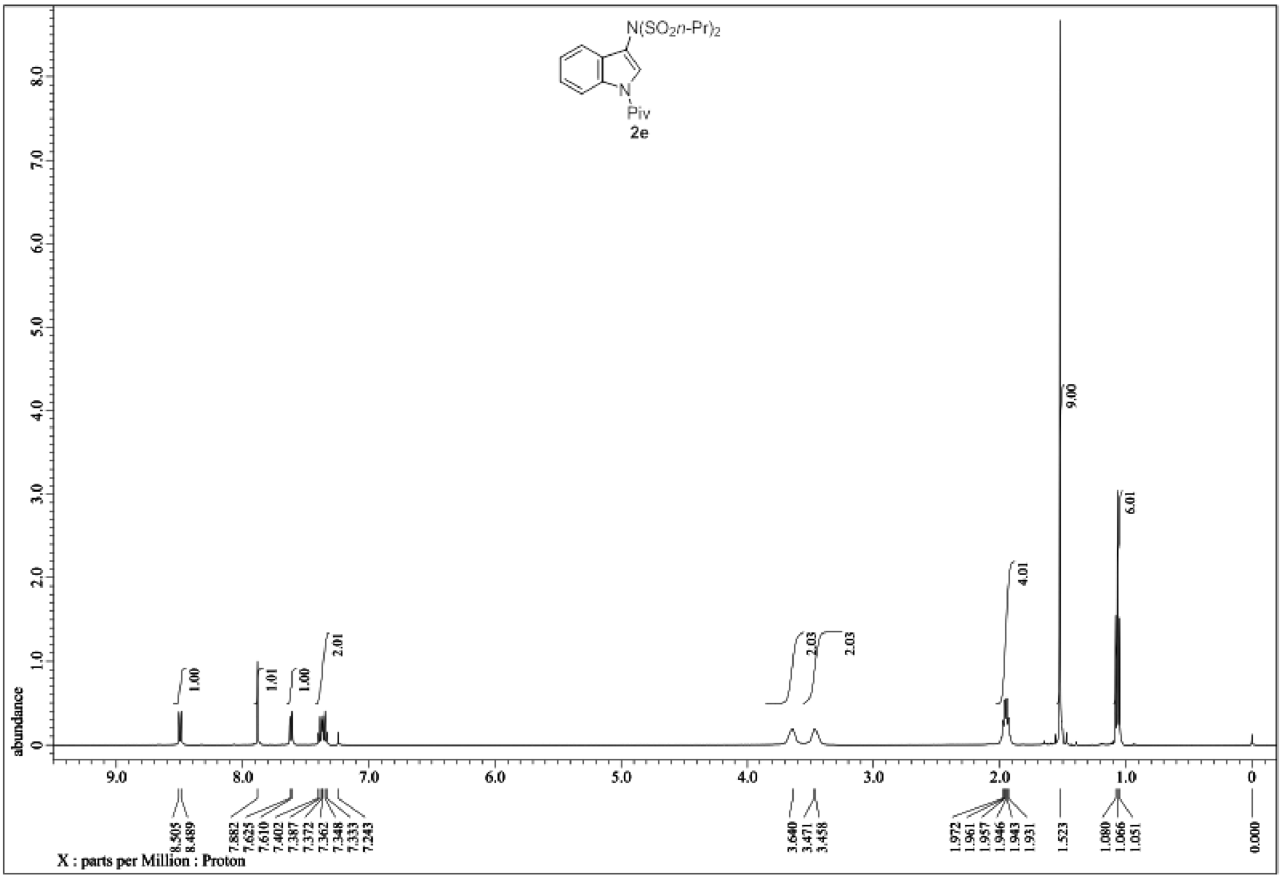
N(SO2n-Pr)2
N
Piv
2e
abundance
X : parts per Million : Proton
8.505
8.489
7.882
7.625
7.610
7.402
7.387
7.372
7.362
7.348
7.333
7.243
3.640
3.471
3.458
1.972
1.961
1.957
1.946
1.943
1.931
1.523
1.080
1.066
1.051
0.000
1.00
1.01
1.00
2.01
2.03
2.03
4.01
9.00
6.01

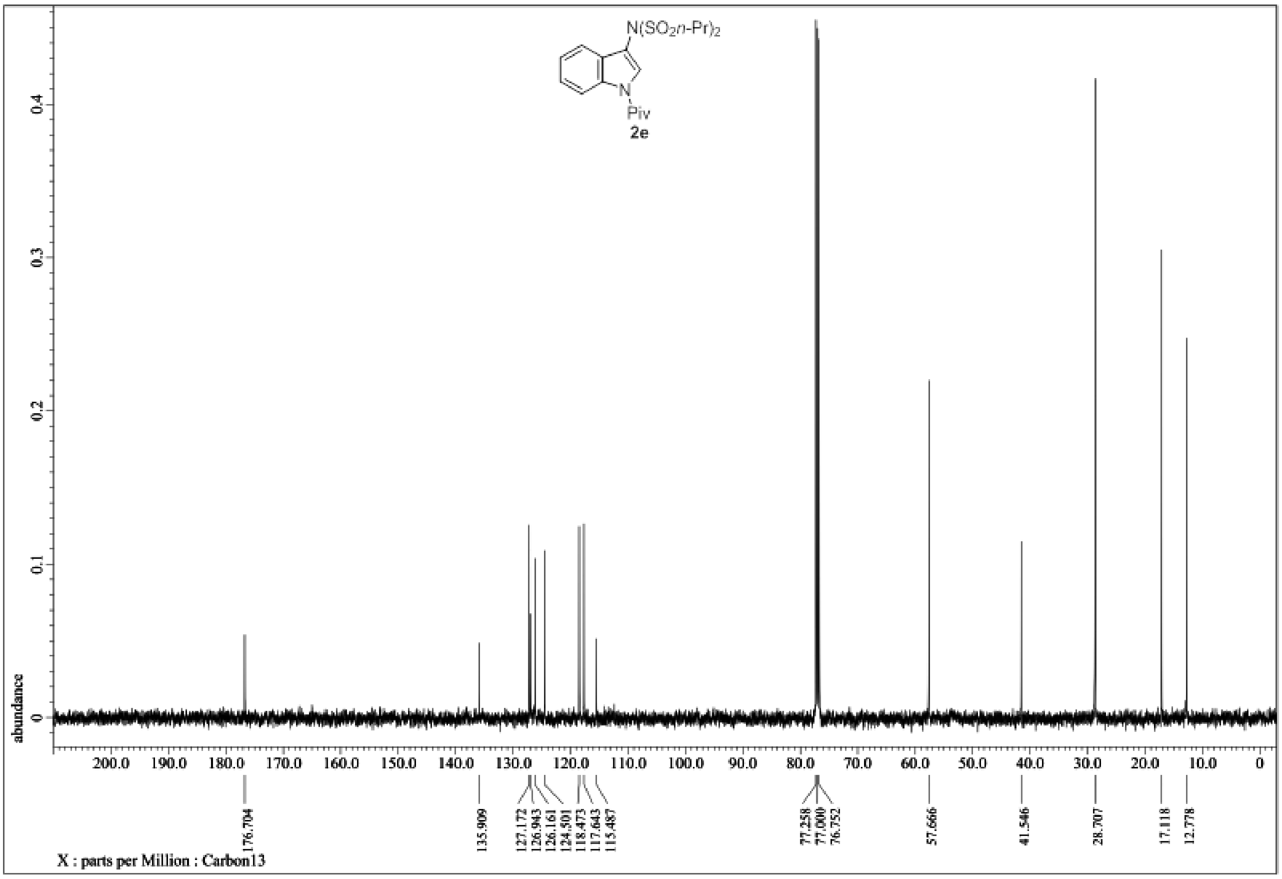
N(SO2n-Pr)2
N
Piv
2e
abundance
X : parts per Million : Carbon13
176.704
135.909
127.172
126.943
126.161
124.501
118.473
117.643
115.487
77.258
77.000
76.752
57.666
41.546
28.707
17.118
12.778

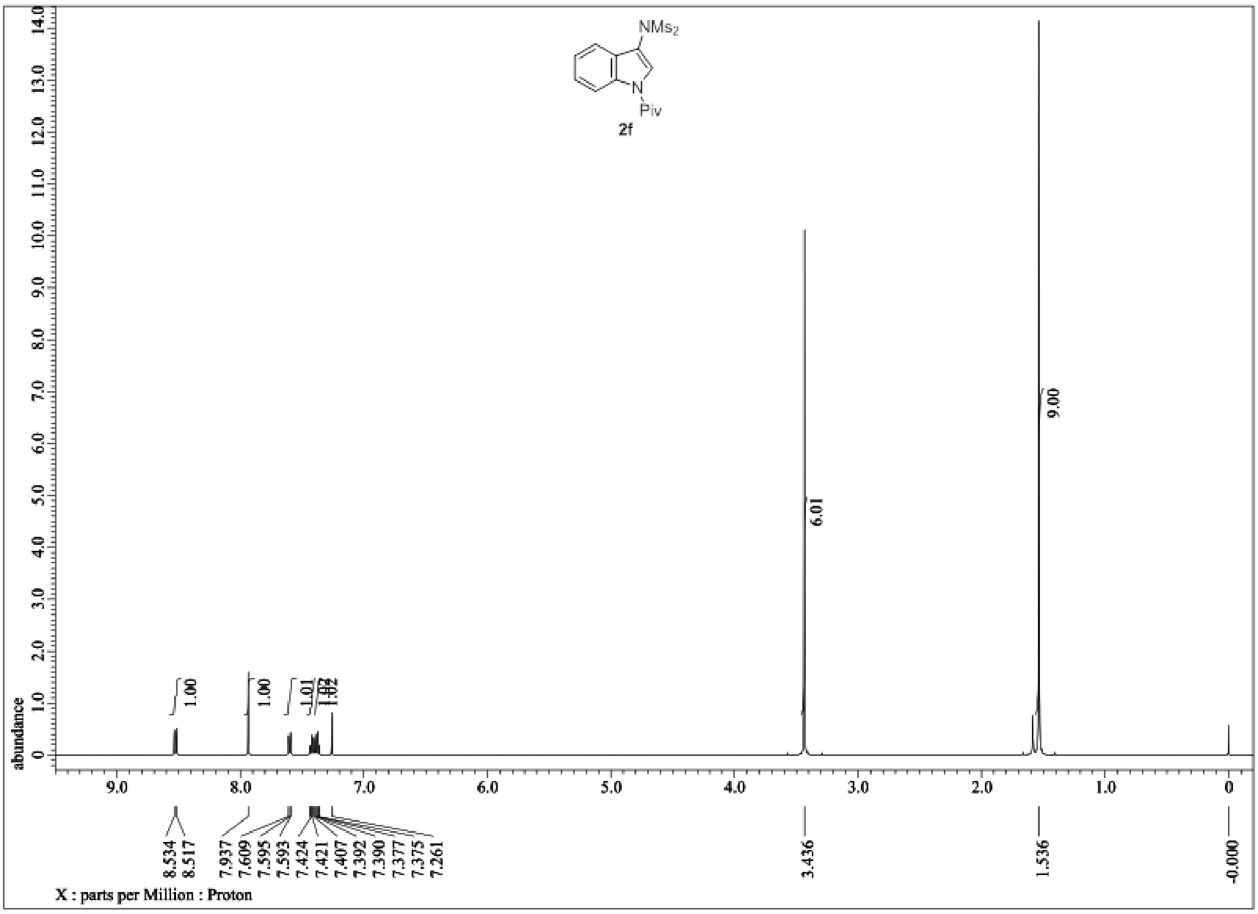
NMs2
N
Piv
2f
abundance
X : parts per Million : Proton
8.534
8.517
7.937
7.609
7.595
7.593
7.424
7.421
7.407
7.392
7.390
7.377
7.375
7.261
3.436
1.536
-0.000
1.00
1.00
1.01
6.01
9.00

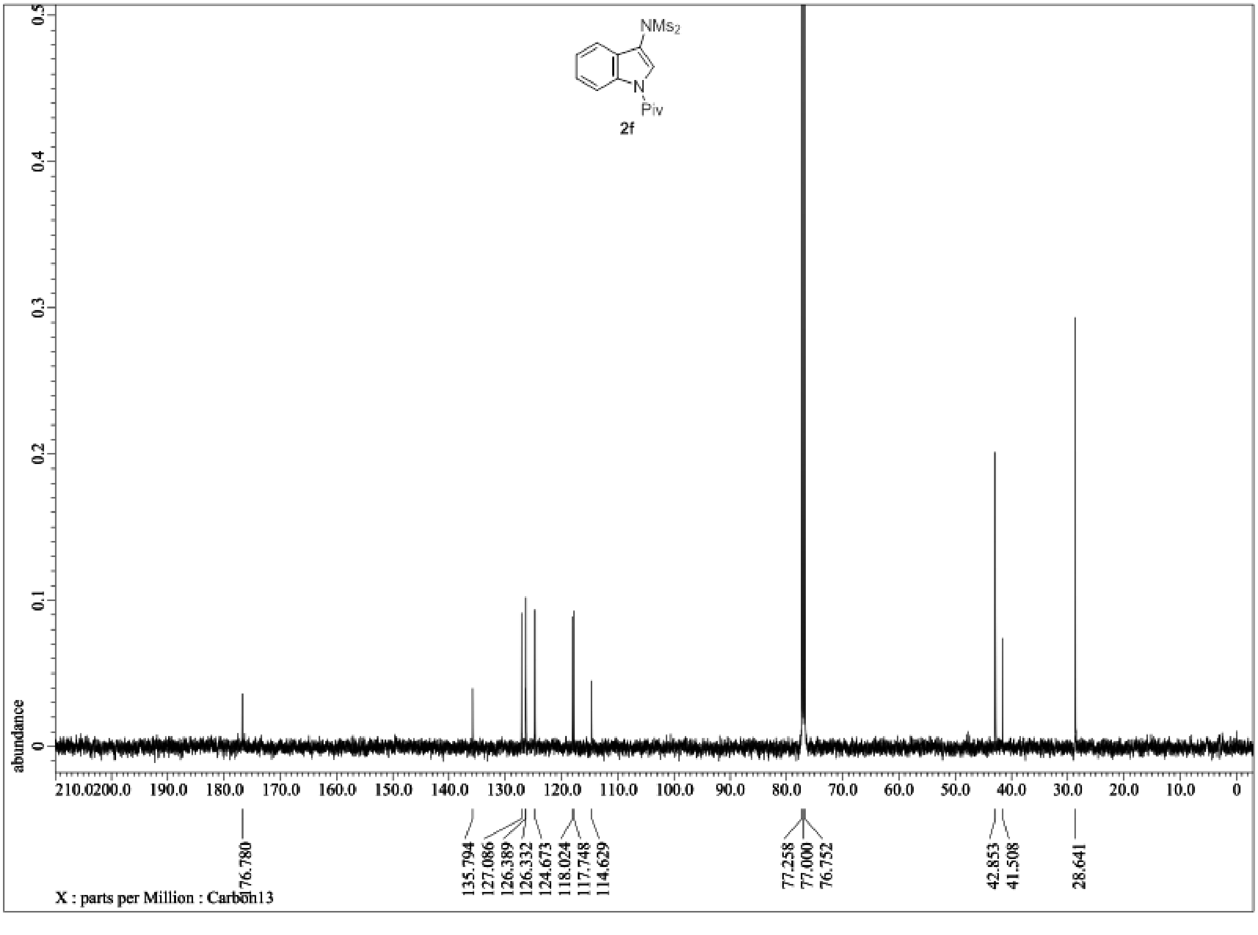
NMs2
N
Piv
2f
abundance
X : parts per Million : Carbon13
176.780
135.794
127.086
126.389
126.332
124.673
118.024
117.748
114.629
77.258
77.000
76.752
42.853
41.508
28.641

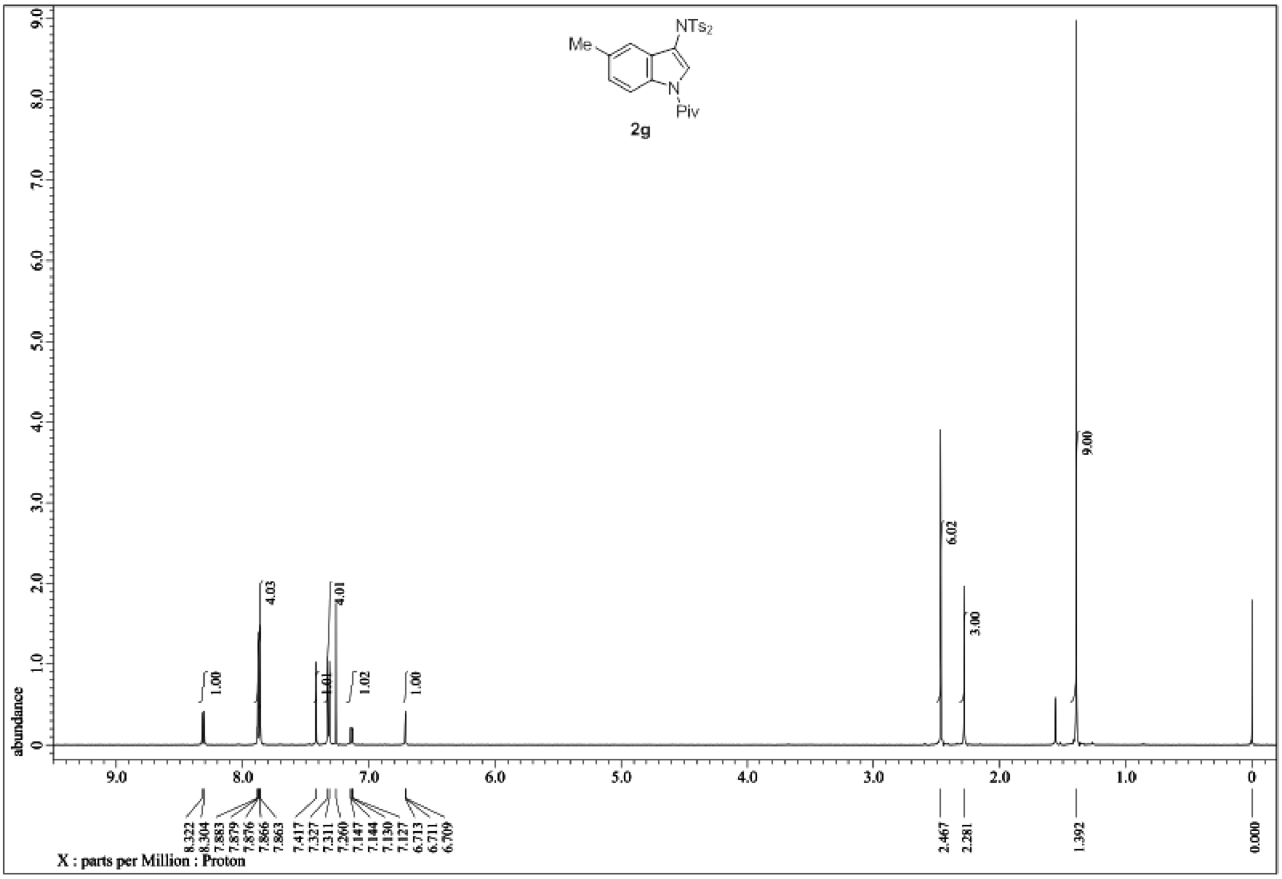
NTs2
Me
N
Piv
2g
abundance
X : parts per Million : Proton

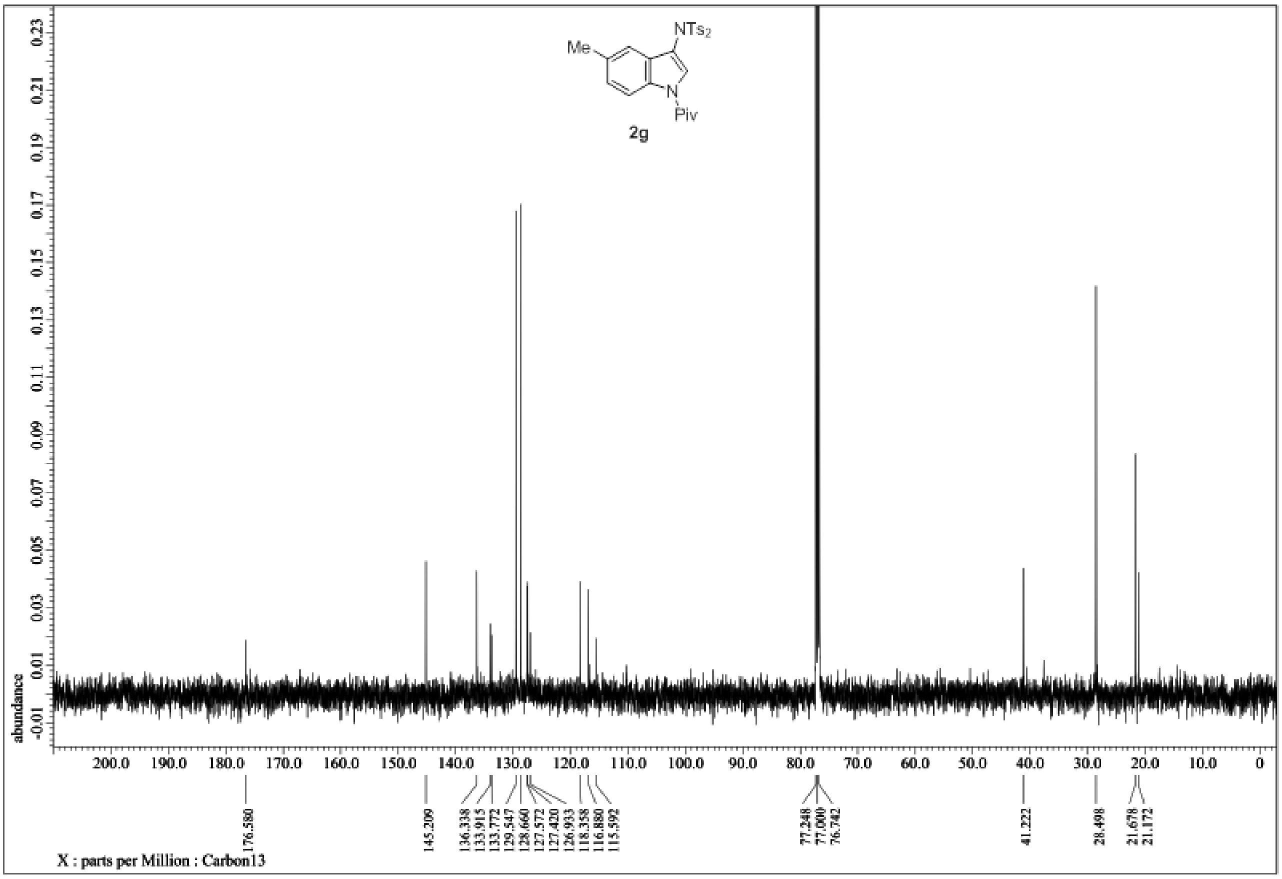
NTs2
Me
N
Piv
2g
abundance
X : parts per Million : Carbon13

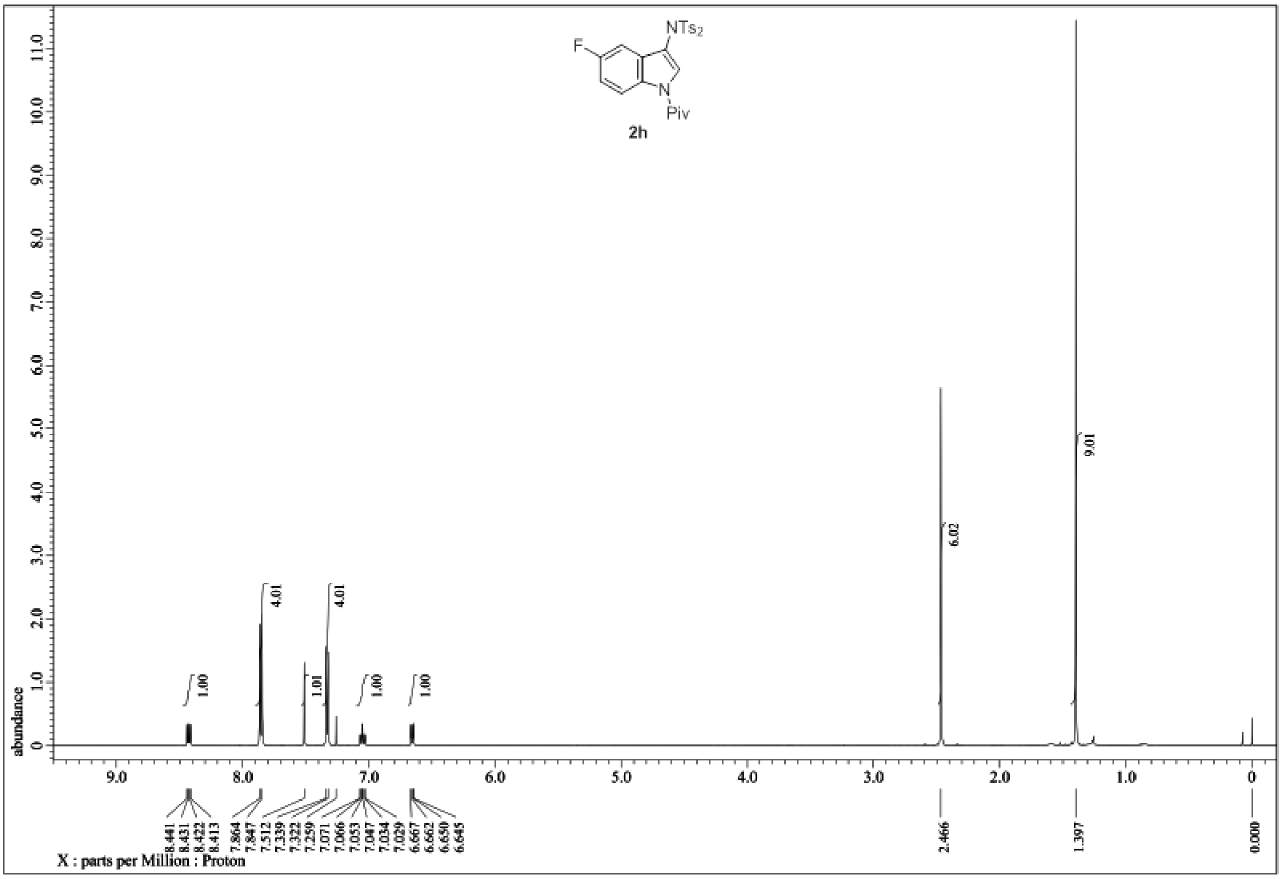
NTs2
F
N
Piv
2h
abundance
X : parts per Million : Proton
1.00
4.01
1.01
4.01
1.00
1.00
6.02
9.01
8.441
8.431
8.422
8.413
7.864
7.847
7.512
7.339
7.322
7.259
7.071
7.066
7.053
7.047
7.034
7.029
6.667
6.662
6.650
6.645
2.466
1.397
0.000

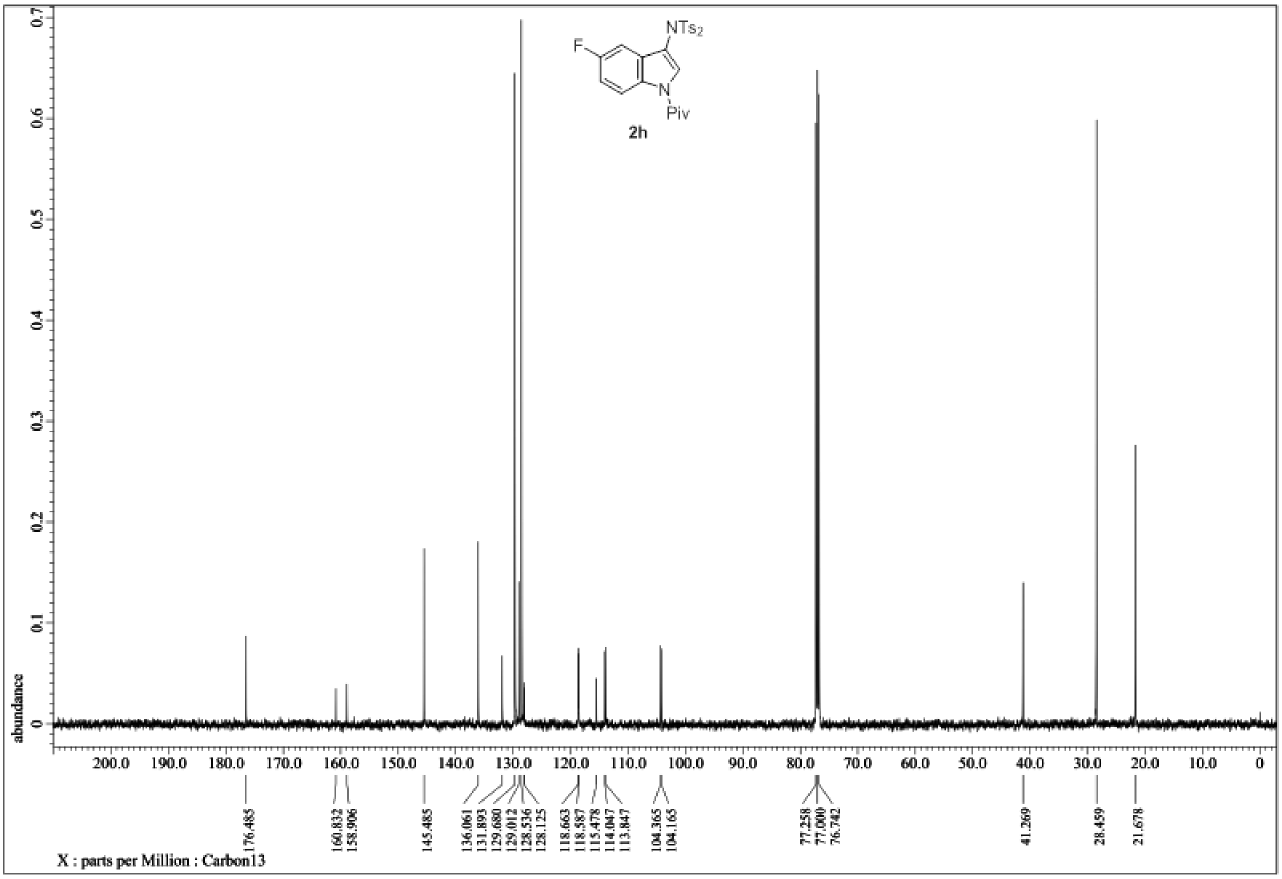
NTs2
F
N
Piv
2h
abundance
X : parts per Million : Carbon13
176.485
160.832
158.906
145.485
136.061
131.893
129.680
129.012
128.536
128.125
118.663
118.587
115.478
114.047
113.847
104.365
104.165
77.258
77.000
76.742
41.269
28.459
21.678

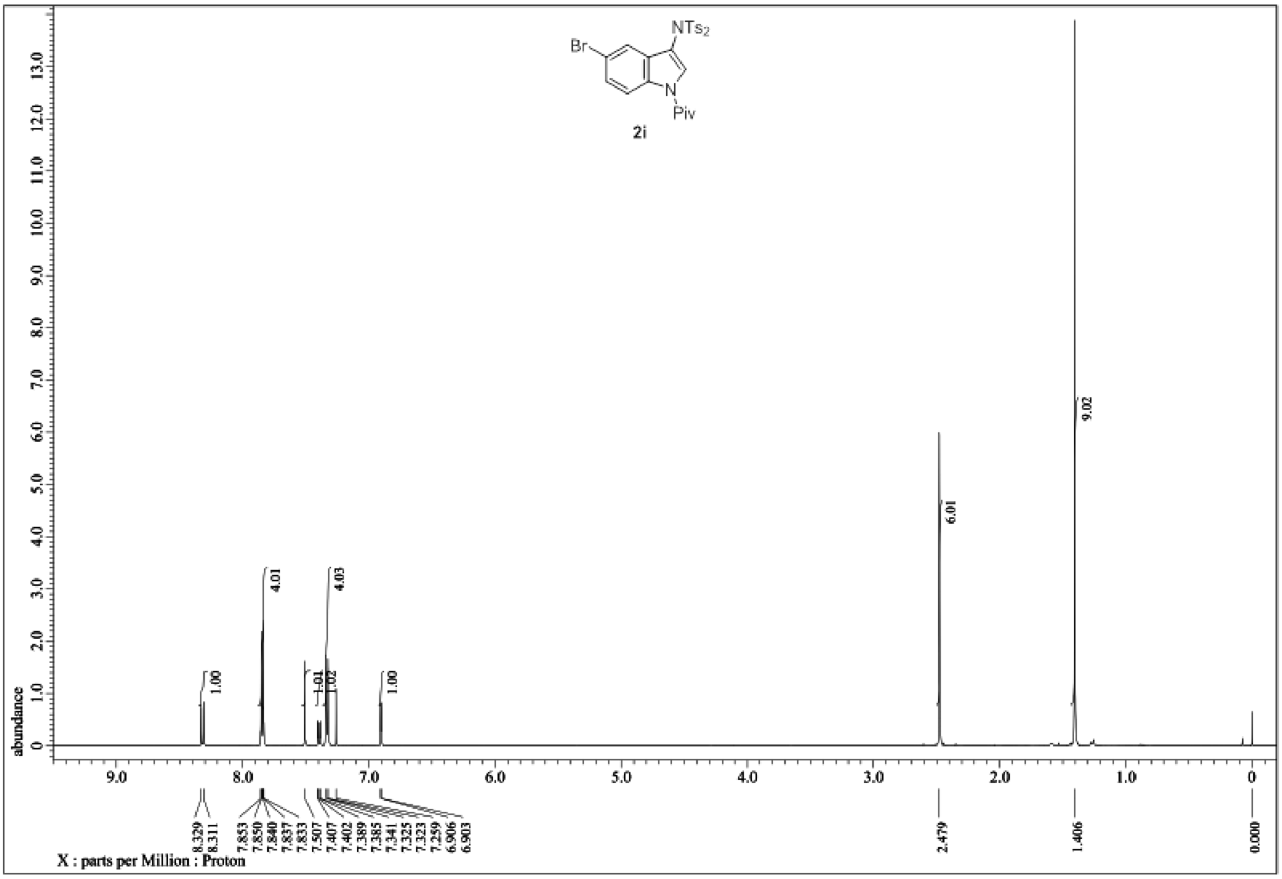

Br
NTs_2
N
Piv
2i
abundance
X : parts per Million : Proton
1.00
4.01
1.01
1.02
4.03
1.00
6.01
9.02
8.329
8.311
7.853
7.850
7.840
7.837
7.833
7.507
7.407
7.402
7.389
7.385
7.341
7.325
7.323
7.259
6.906
6.903
2.479
1.406
0.000

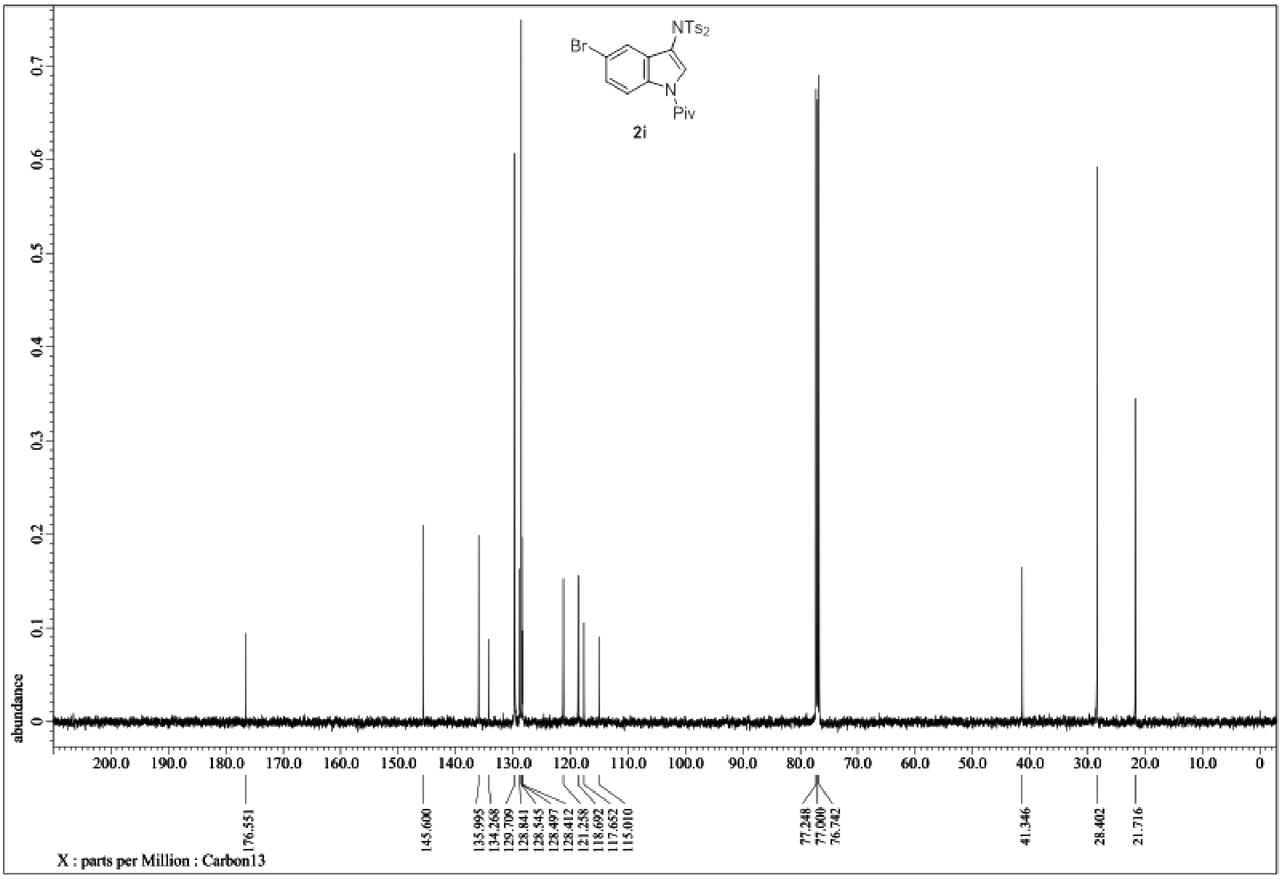

Br
NTs_2
N
Piv
2i
abundance
X : parts per Million : Carbon13
176.551
145.600
135.995
134.268
129.709
128.841
128.545
128.497
128.412
121.258
118.692
117.652
115.010
77.248
77.000
76.742
41.346
28.402
21.716

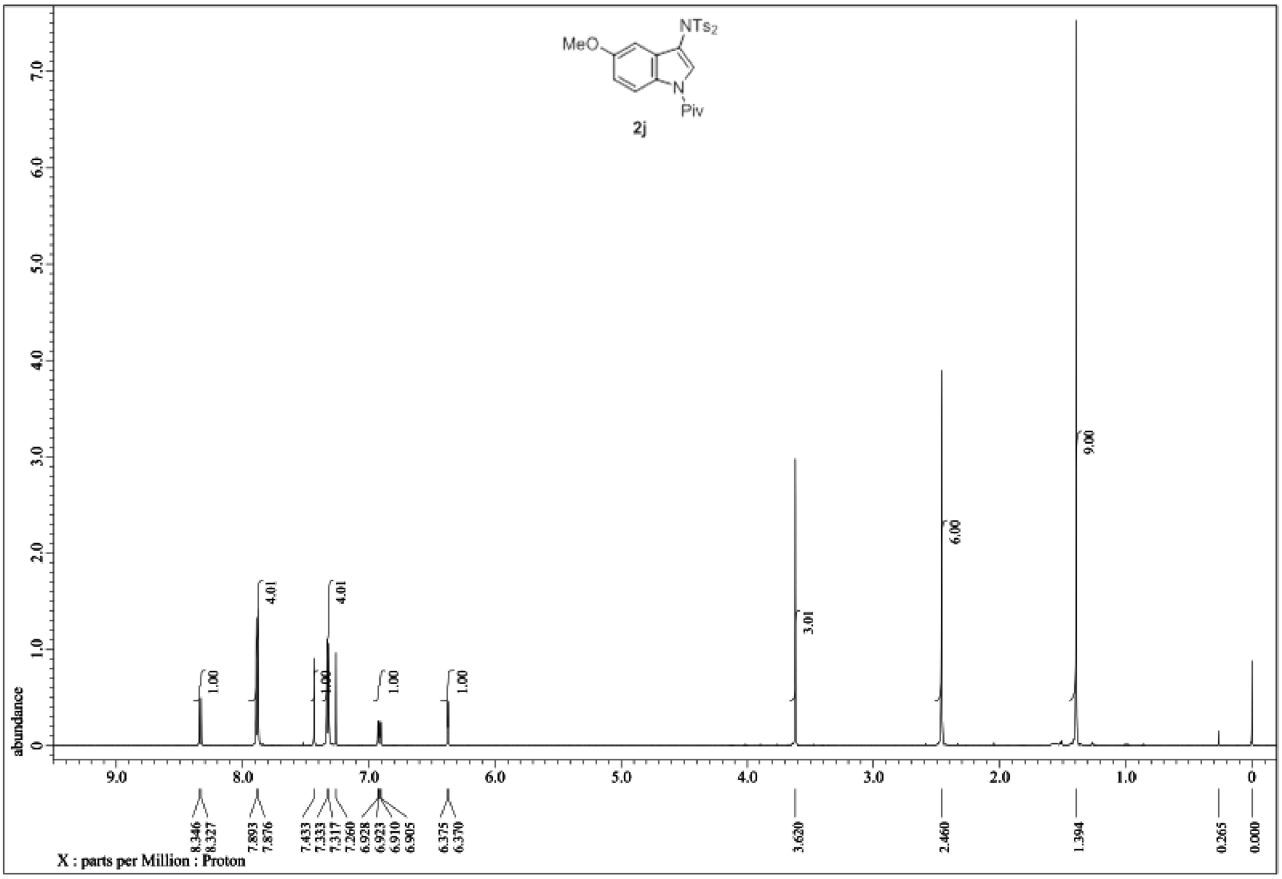
MeO
NTs2
N
Piv
2j
abundance
X : parts per Million : Proton
8.346 8.327 7.893 7.876 7.433 7.333 7.317 7.260 6.928 6.923 6.910 6.905 6.375 6.370 3.620 2.460 1.394 0.265 0.000
1.00 4.01 1.00 4.01 1.00 1.00 3.01 6.00 9.00

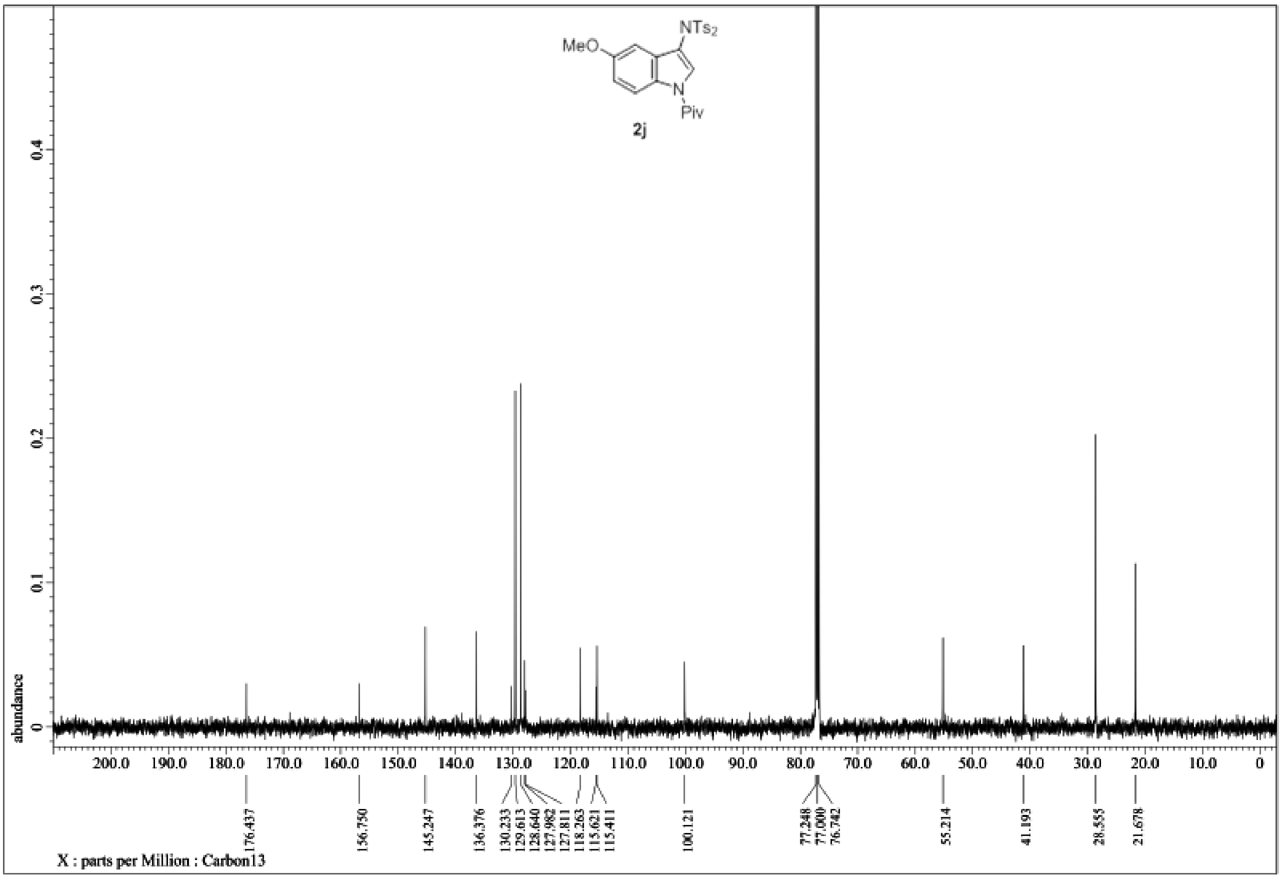
MeO
NTs2
N
Piv
2j
abundance
X : parts per Million : Carbon13
176.437 156.750 145.247 136.376 130.233 129.613 128.640 127.982 127.811 118.263 115.621 115.411 100.121 77.248 77.000 76.742 55.214 41.193 28.555 21.678

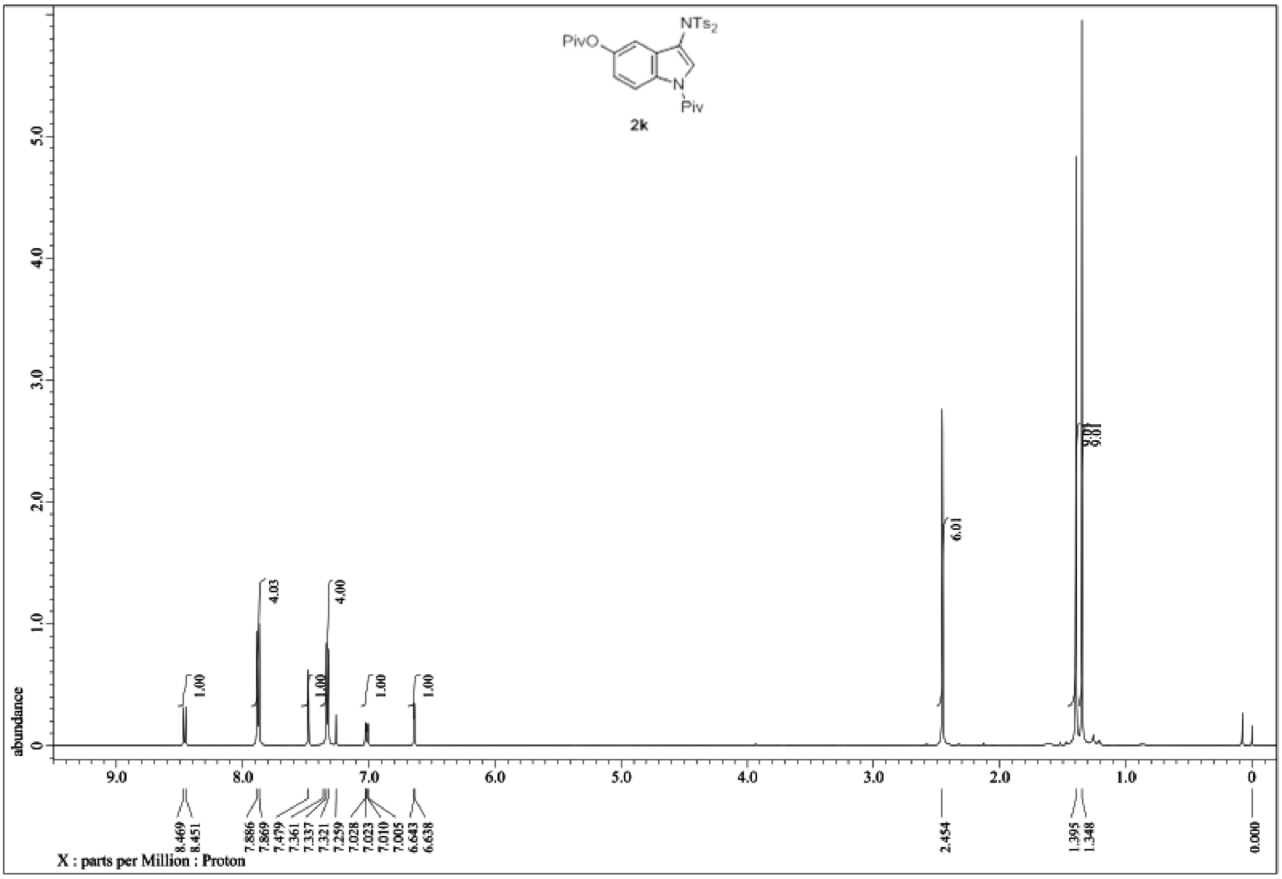

PivO
NTs2
N
Piv
2k
abundance
X : parts per Million : Proton
8.469
8.451
7.886
7.869
7.479
7.361
7.337
7.321
7.259
7.028
7.023
7.010
7.005
6.643
6.638
2.454
1.395
1.348
0.000
1.00
4.03
1.00
4.00
1.00
1.00
6.01
9.01
9.01

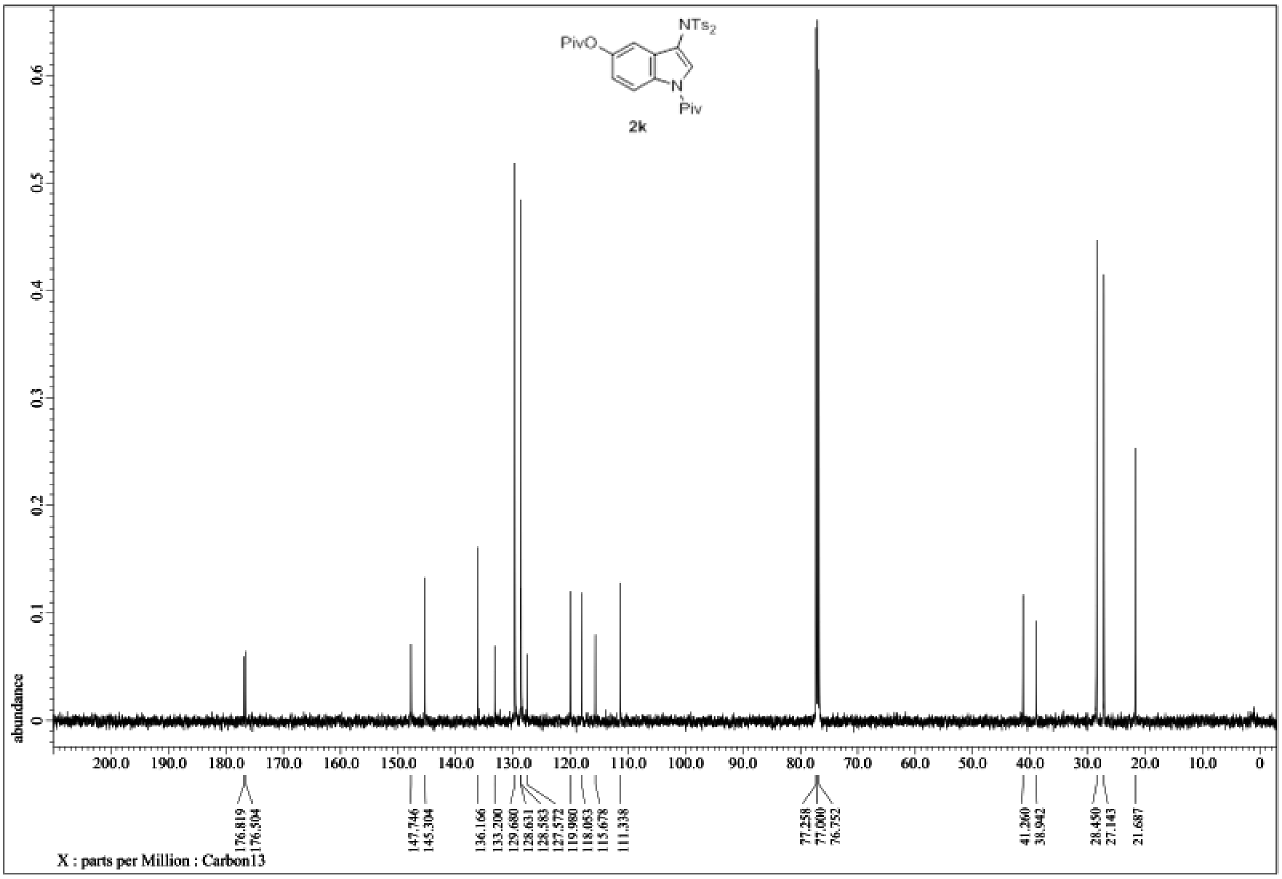

PivO
NTs2
N
Piv
2k
abundance
X : parts per Million : Carbon13
176.819
176.504
147.746
145.304
136.166
133.200
129.680
128.631
128.583
127.572
119.980
118.053
115.678
111.338
77.258
77.000
76.752
41.260
38.942
28.450
27.143
21.687

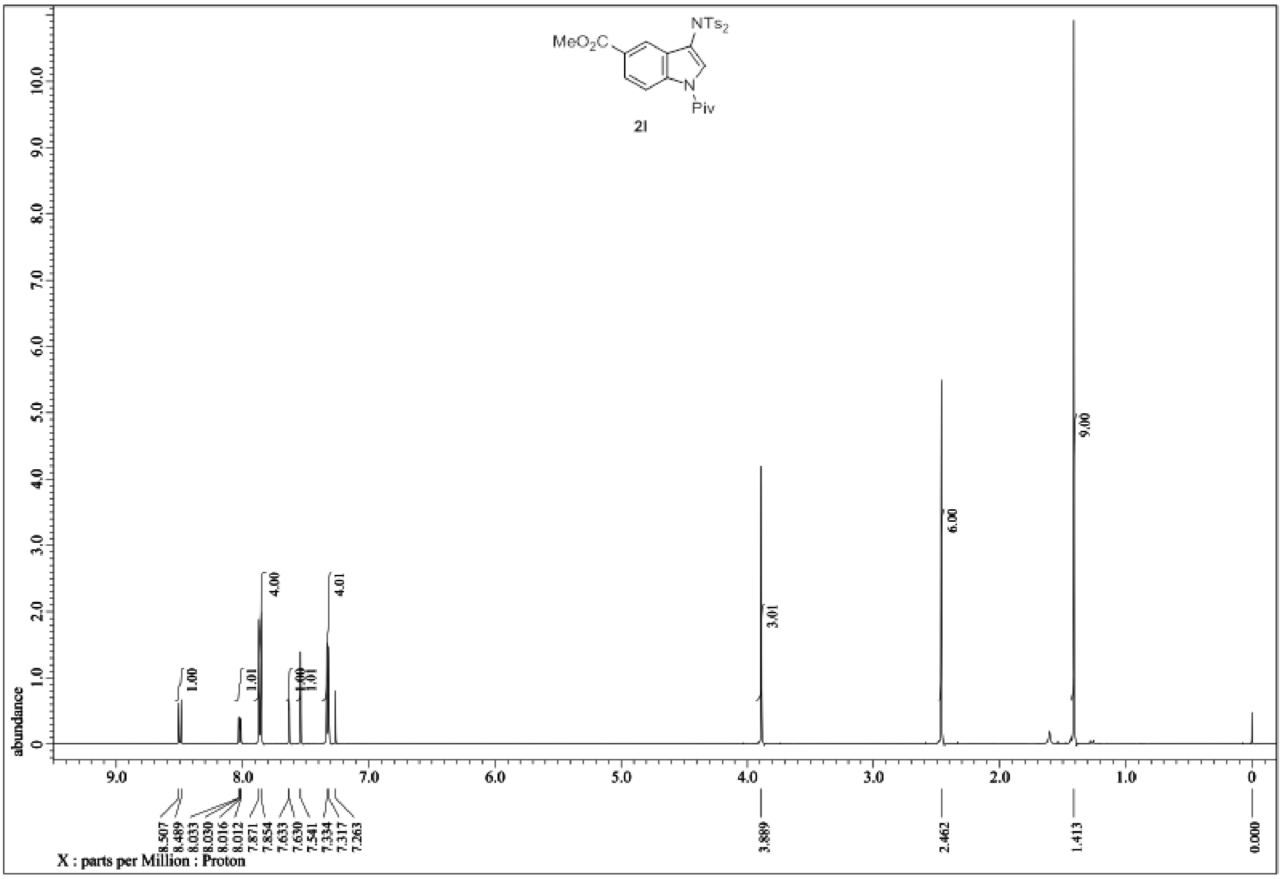

MeO2C
NTs2
N
Piv
2l
abundance
X : parts per Million : Proton
8.507
8.489
8.033
8.030
8.016
8.012
7.871
7.854
7.633
7.630
7.541
7.334
7.317
7.263
3.889
2.462
1.413
0.000
1.00
1.01
4.00
1.00
1.01
4.01
3.01
6.00
9.00

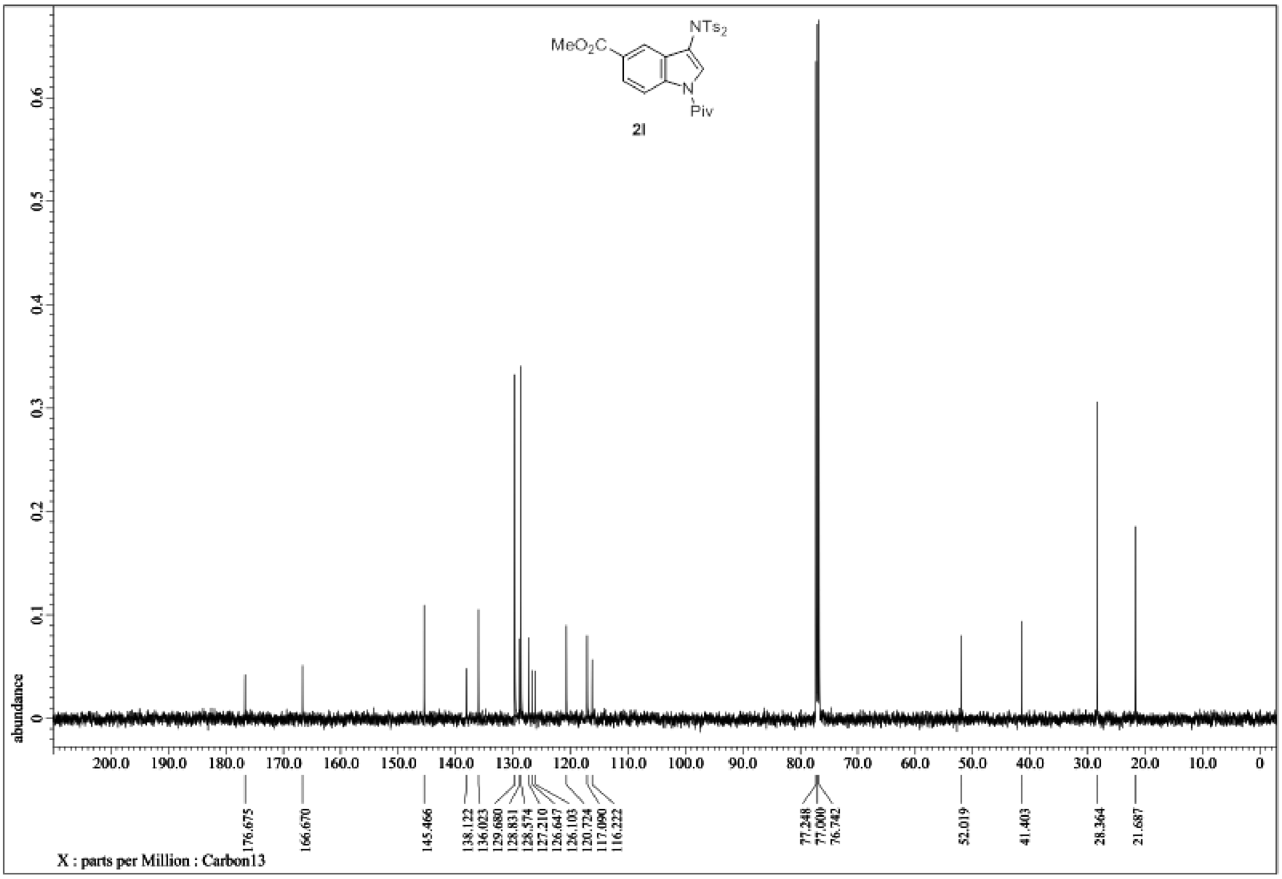

MeO2C
NTs2
N
Piv
2l
abundance
X : parts per Million : Carbon13
176.675
166.670
145.466
138.122
136.023
129.680
128.831
128.574
127.210
126.647
126.103
120.724
117.090
116.222
77.248
77.000
76.742
52.019
41.403
28.364
21.687

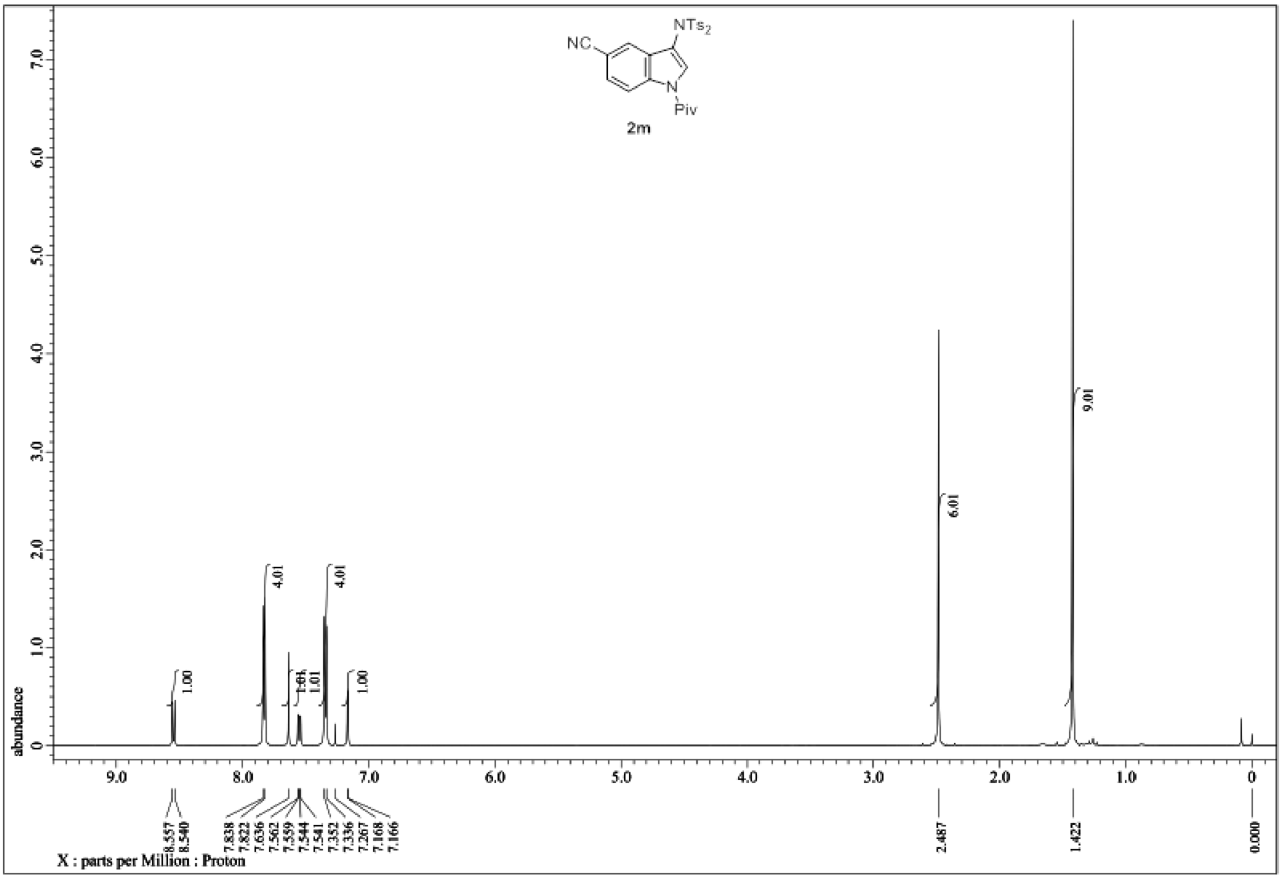
NTs2
NC
N
Piv
2m
X : parts per Million : Proton
abundance
8.557
8.540
7.838
7.822
7.636
7.562
7.559
7.544
7.541
7.352
7.336
7.267
7.168
7.166
2.487
1.422
0.000
1.00
4.01
1.01
1.01
4.01
1.00
6.01
9.01

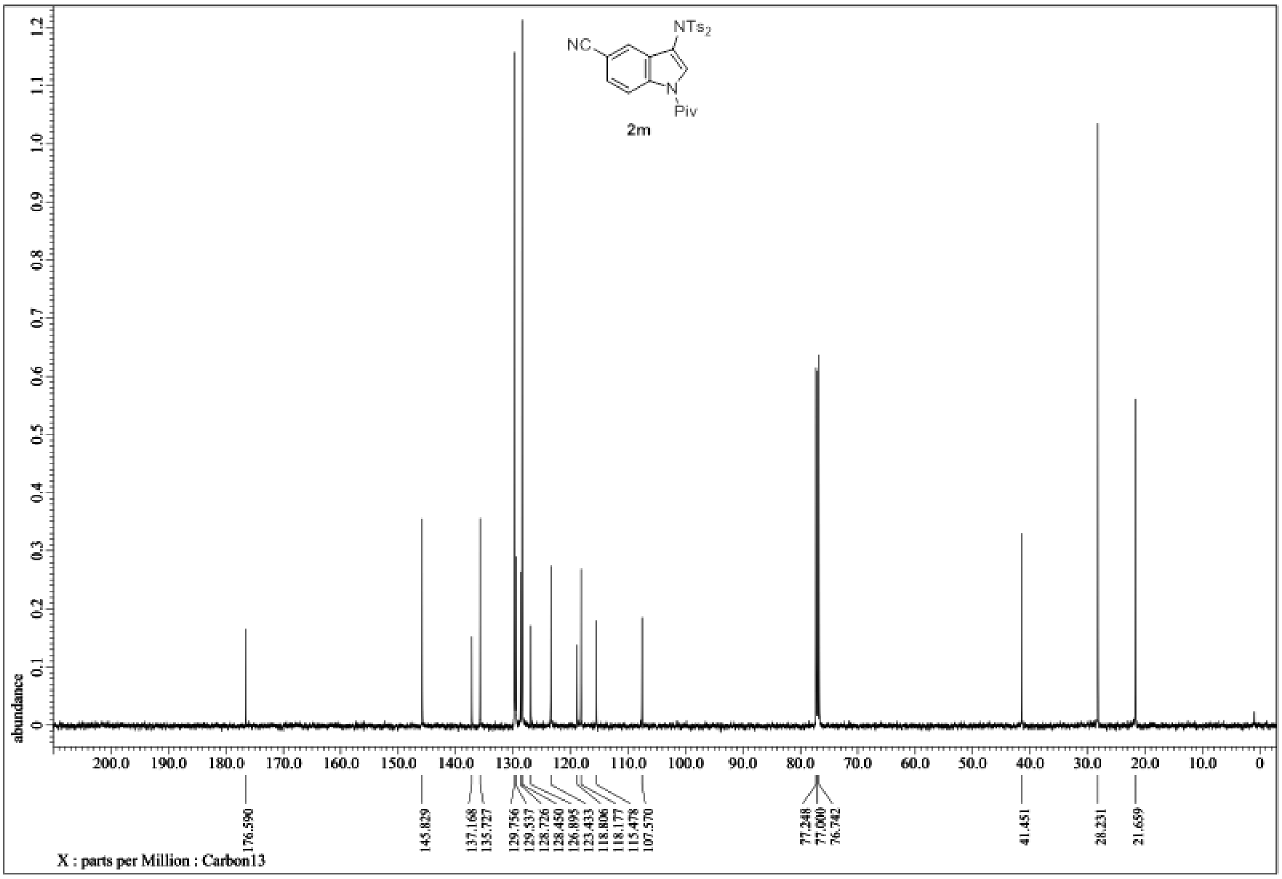
NTs2
NC
N
Piv
2m
X : parts per Million : Carbon13
abundance
176.590
145.829
137.168
135.727
129.756
129.537
128.726
128.450
126.895
123.433
118.806
118.177
115.478
107.570
77.248
77.000
76.742
41.451
28.231
21.659

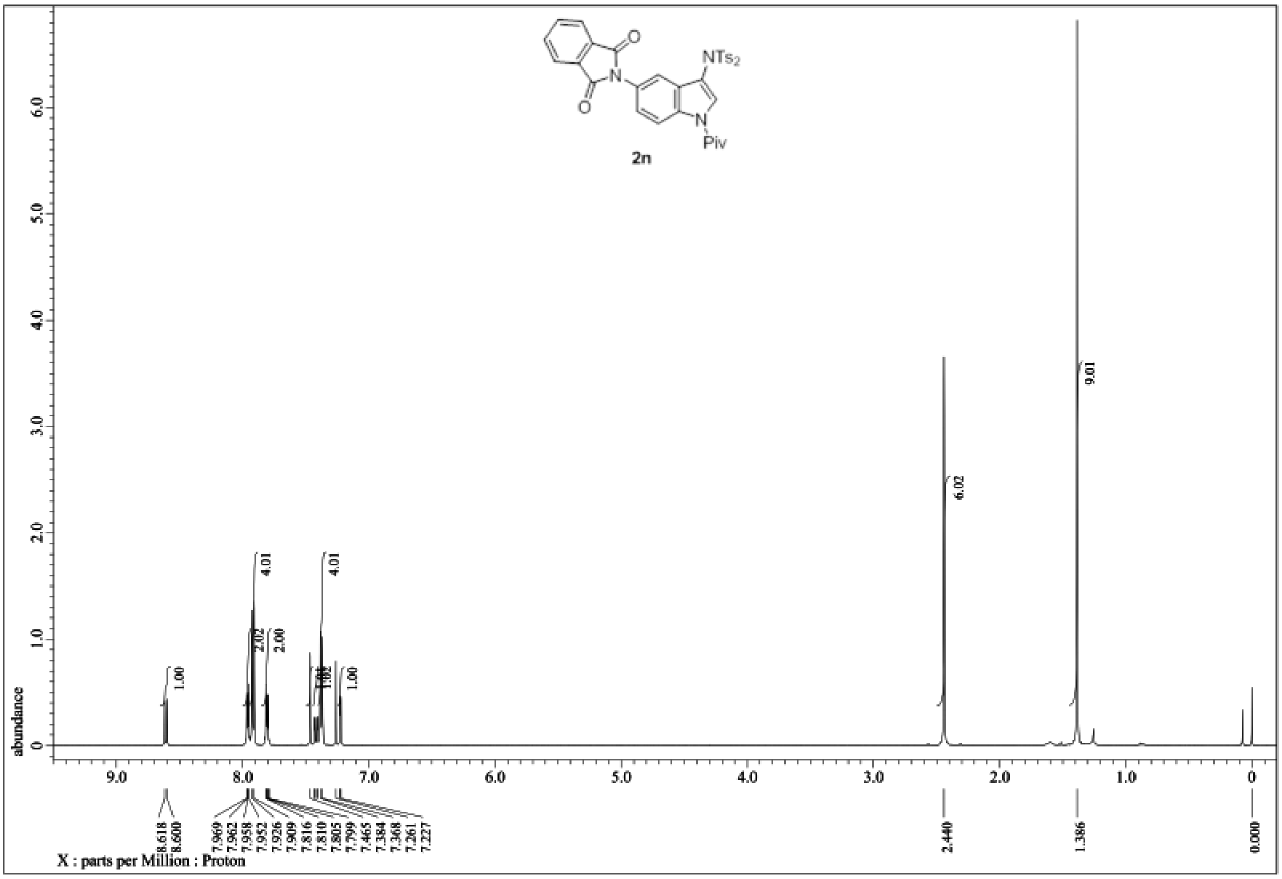
NTs2
N
O
Piv
2n
abundance
9.01
6.02
4.01
4.01
2.02
2.00
1.00
1.00
8.618
8.600
7.969
7.962
7.958
7.952
7.926
7.909
7.816
7.810
7.805
7.799
7.465
7.384
7.368
7.261
7.227
2.440
1.386
0.000
X : parts per Million : Proton

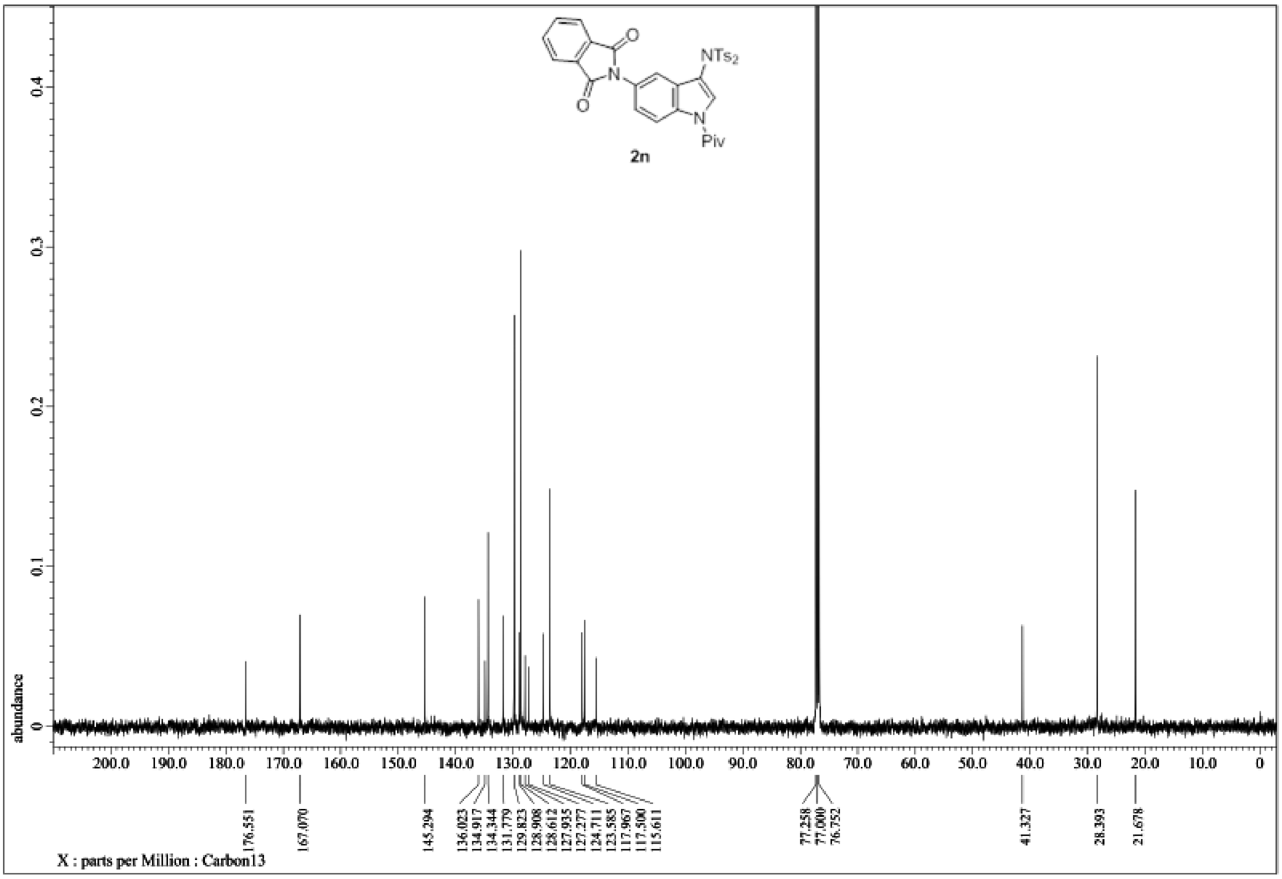
NTs2
N
O
Piv
2n
abundance
176.551
167.070
145.294
136.023
134.917
134.344
131.779
129.823
128.908
128.612
127.935
127.277
124.711
123.585
117.967
117.500
115.611
77.258
77.000
76.752
41.327
28.393
21.678
X : parts per Million : Carbon13

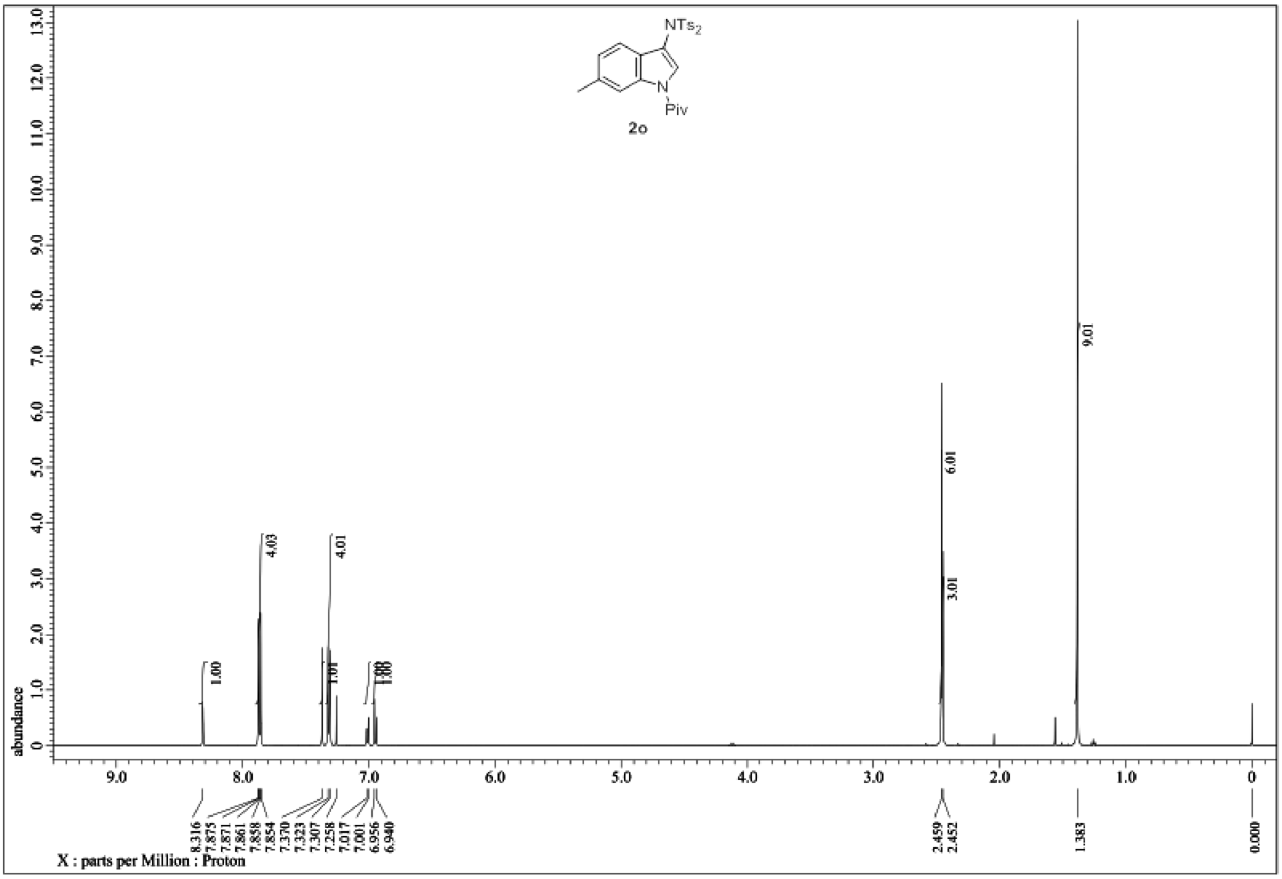
NTs2
Piv
2o
abundance
X : parts per Million : Proton
8.316
7.875
7.871
7.861
7.858
7.854
7.370
7.323
7.307
7.258
7.017
7.001
6.956
6.940
2.459
2.452
1.383
0.000
1.00
4.03
4.01
1.01
6.01
3.01
9.01

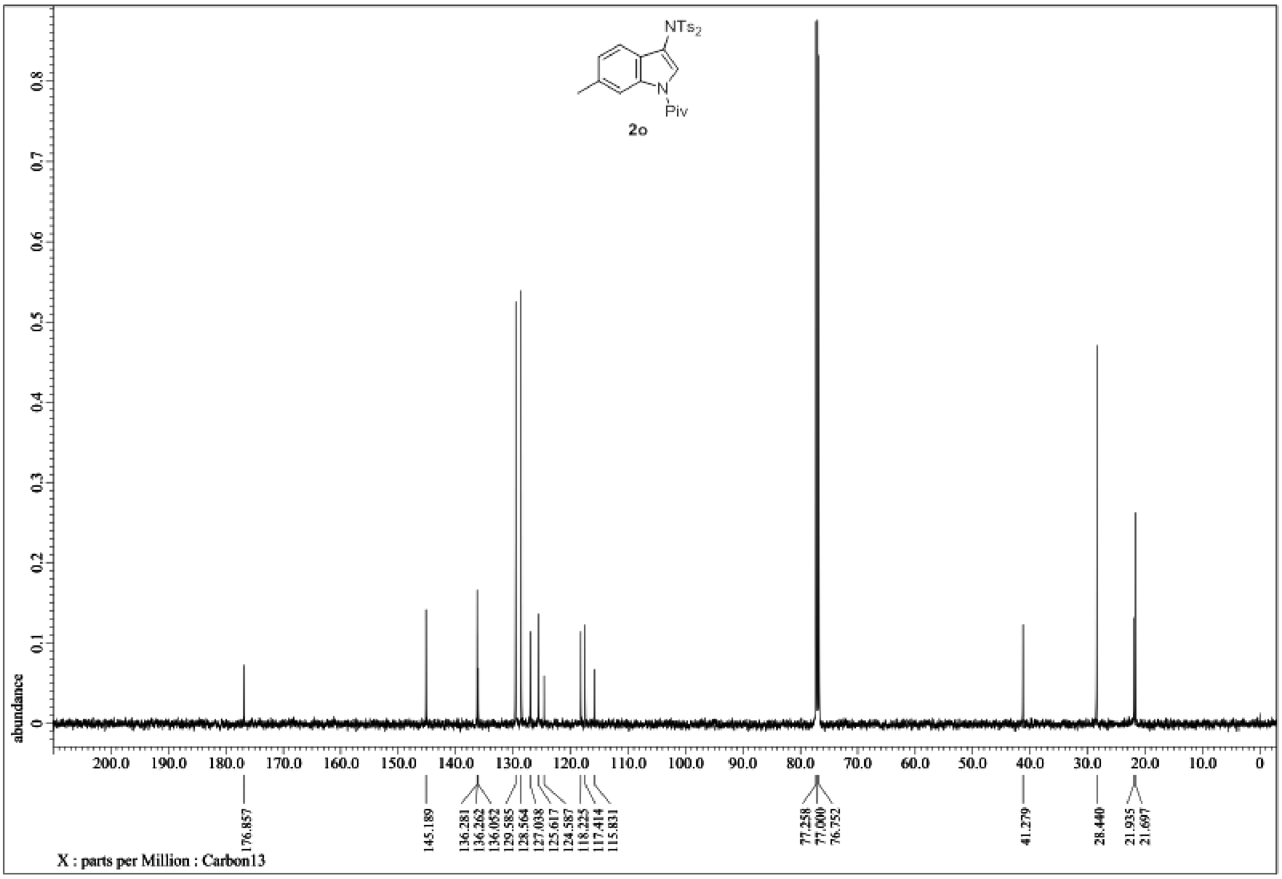
NTs2
Piv
2o
abundance
X : parts per Million : Carbon13
176.857
145.189
136.281
136.262
136.052
129.585
128.564
127.038
125.617
124.587
118.225
117.414
115.831
77.258
77.000
76.752
41.279
28.440
21.935
21.697

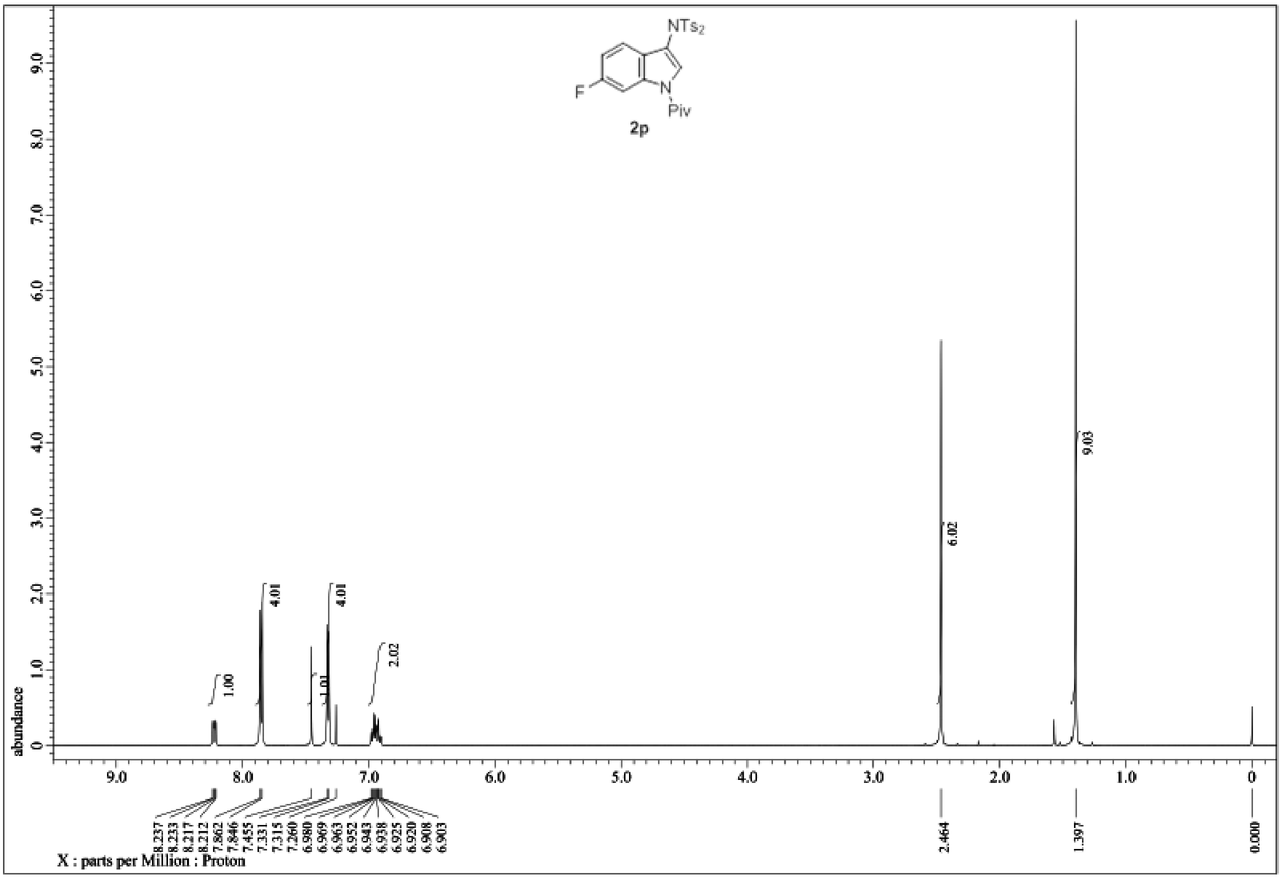
NTs2
F
N
Piv
2p
abundance
X : parts per Million : Proton
1.00
4.01
1.01
4.01
2.02
6.02
9.03
2.464
1.397
0.000

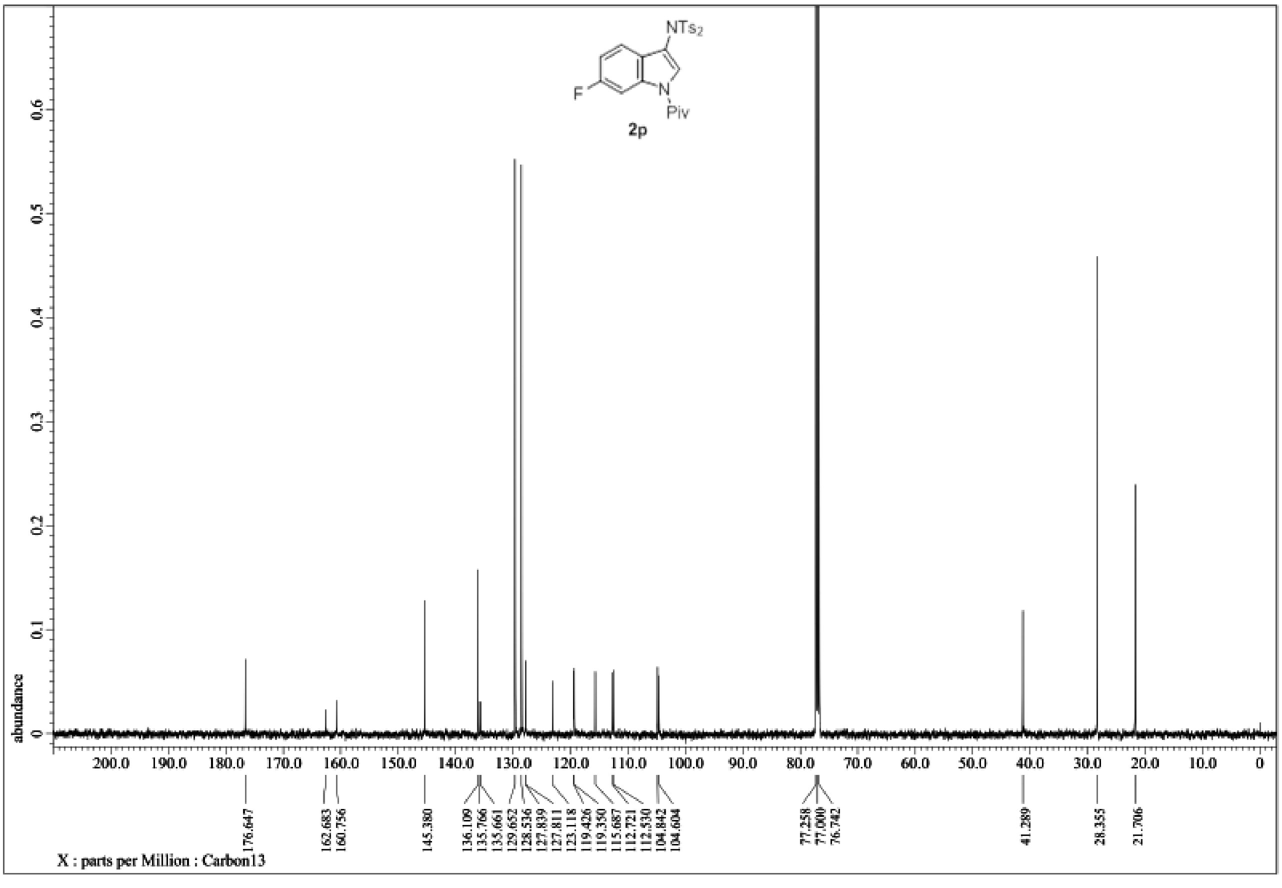
NTs2
F
N
Piv
2p
abundance
X : parts per Million : Carbon13
176.647
162.683
160.756
145.380
77.258
77.000
76.742
41.289
28.355
21.706

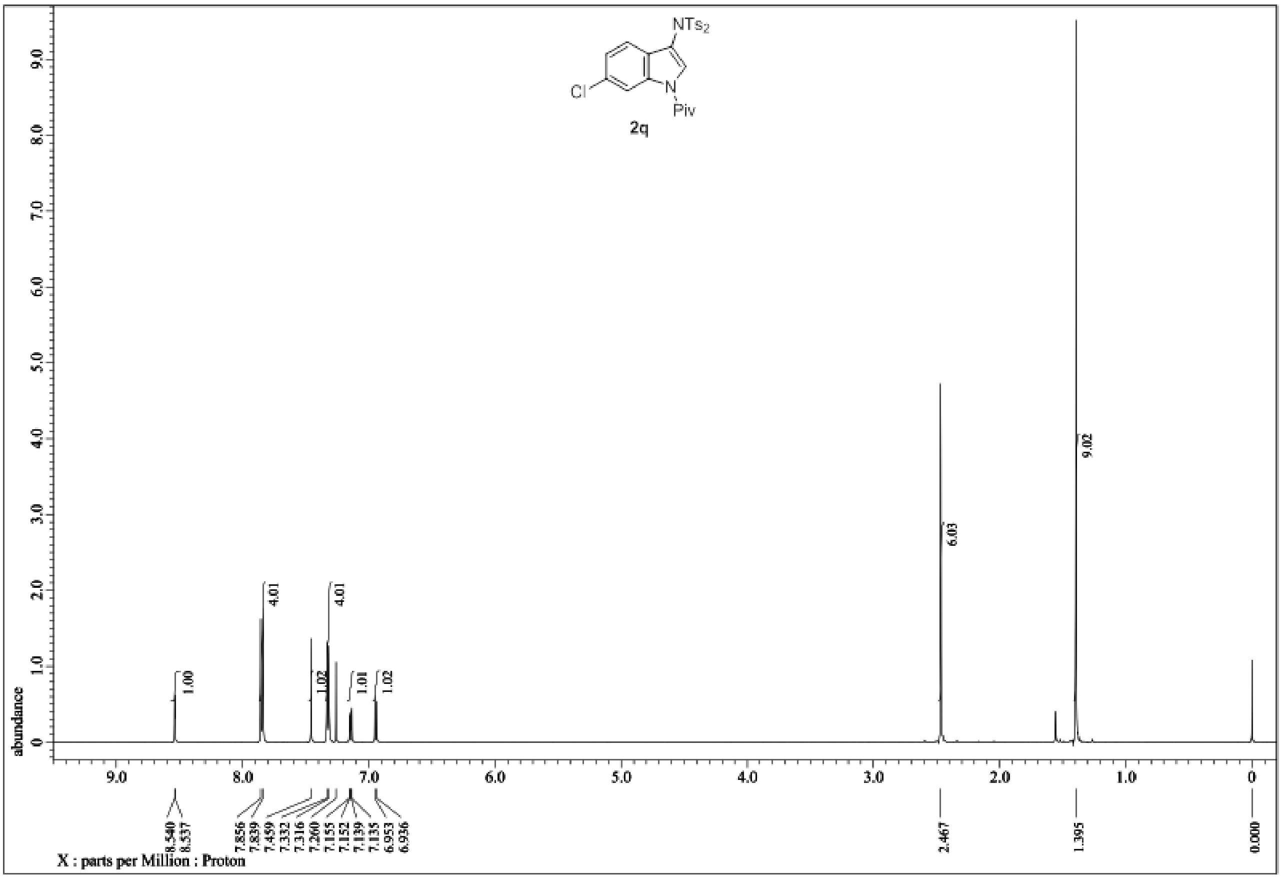
NTs2
Cl
N
Piv
2q
abundance
X : parts per Million : Proton
8.540
8.537
7.856
7.839
7.459
7.332
7.316
7.260
7.155
7.152
7.139
7.135
6.953
6.936
2.467
1.395
0.000
1.00
4.01
1.02
4.01
1.01
1.02
6.03
9.02

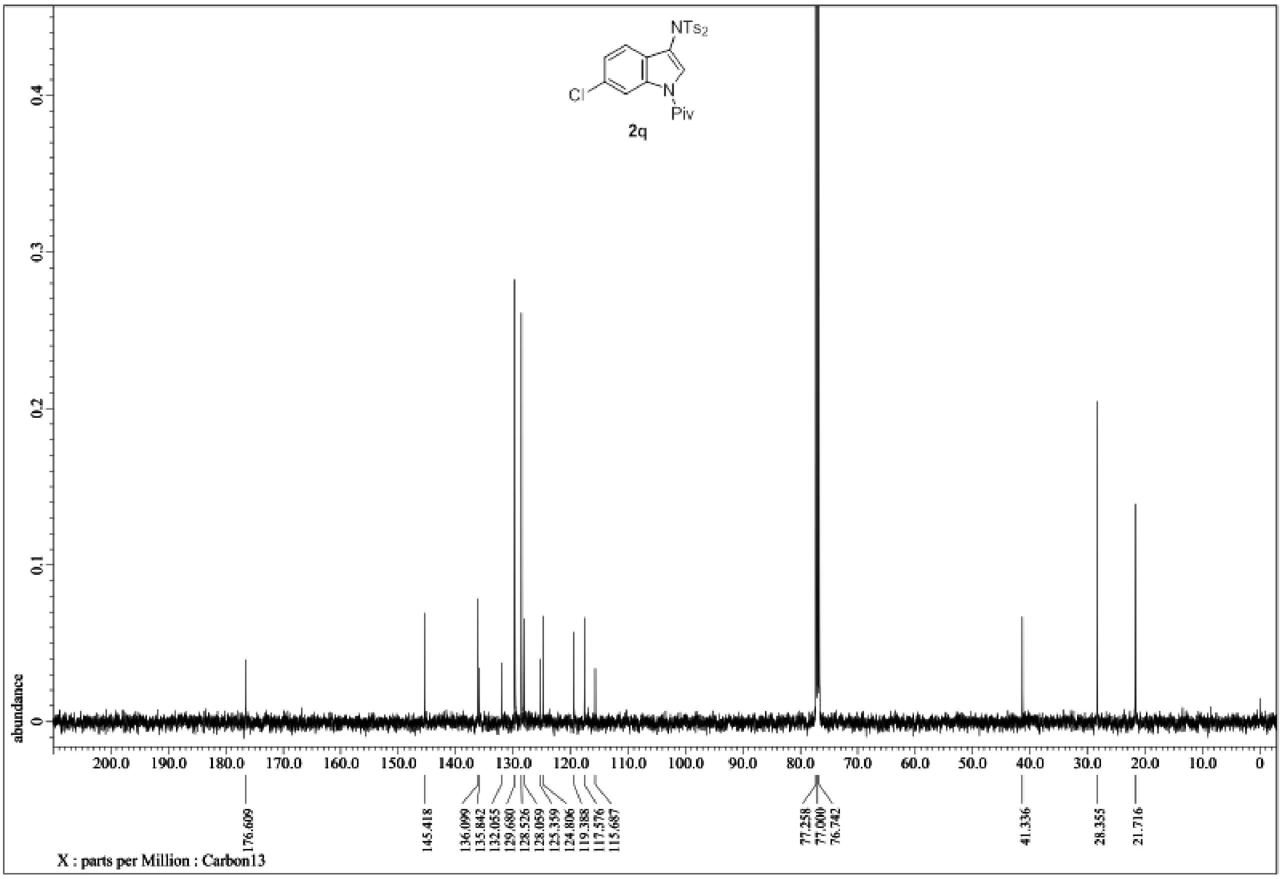
NTs2
Cl
N
Piv
2q
abundance
X : parts per Million : Carbon13
176.609
145.418
136.099
135.842
132.055
129.680
128.526
128.059
125.359
124.806
119.388
117.576
115.687
77.258
77.000
76.742
41.336
28.355
21.716

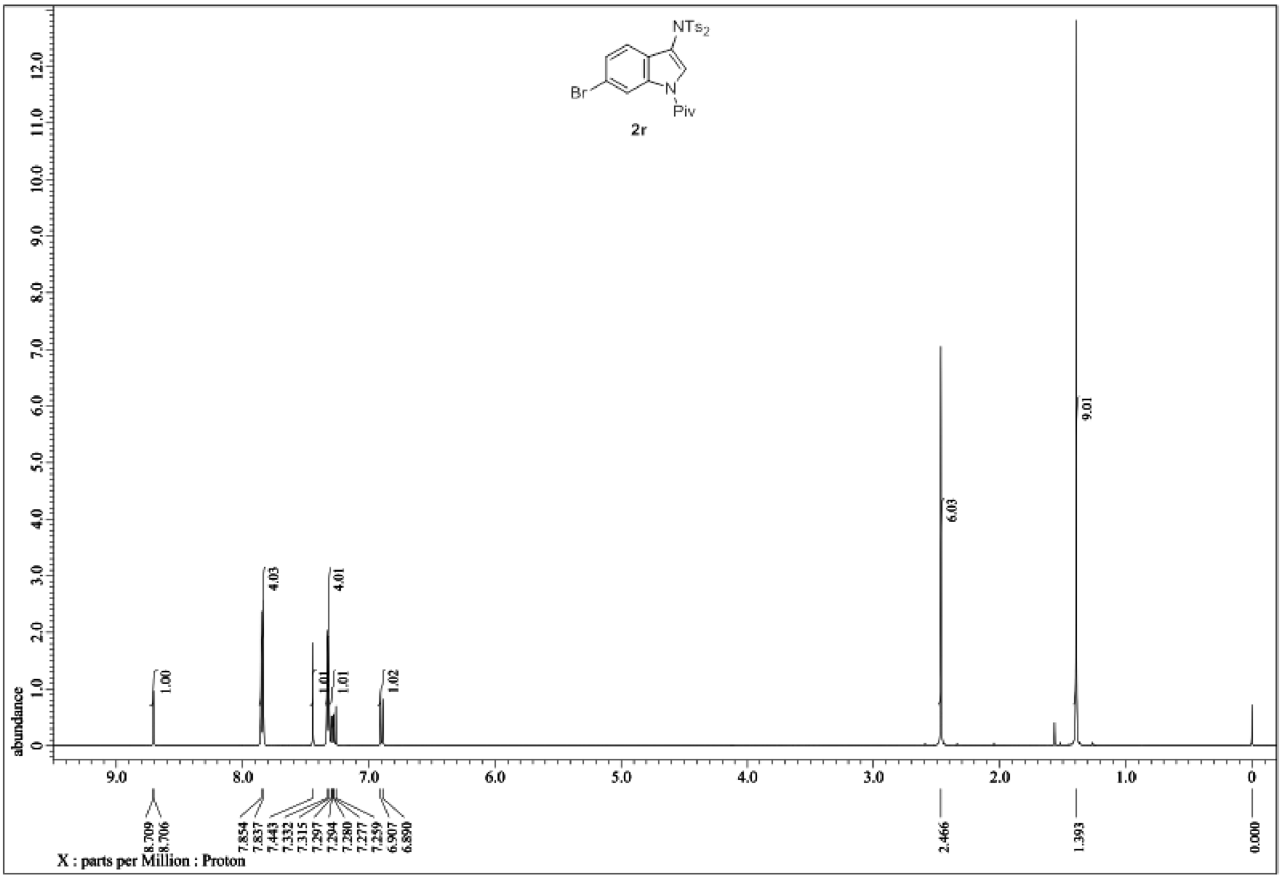
NTs2
Br
N
Piv
2r
abundance
X : parts per Million : Proton
8.709
8.706
7.854
7.837
7.443
7.332
7.315
7.297
7.204
7.280
7.277
7.259
6.907
6.890
2.466
1.393
0.000
1.00
4.03
1.01
4.01
1.01
1.02
6.03
9.01

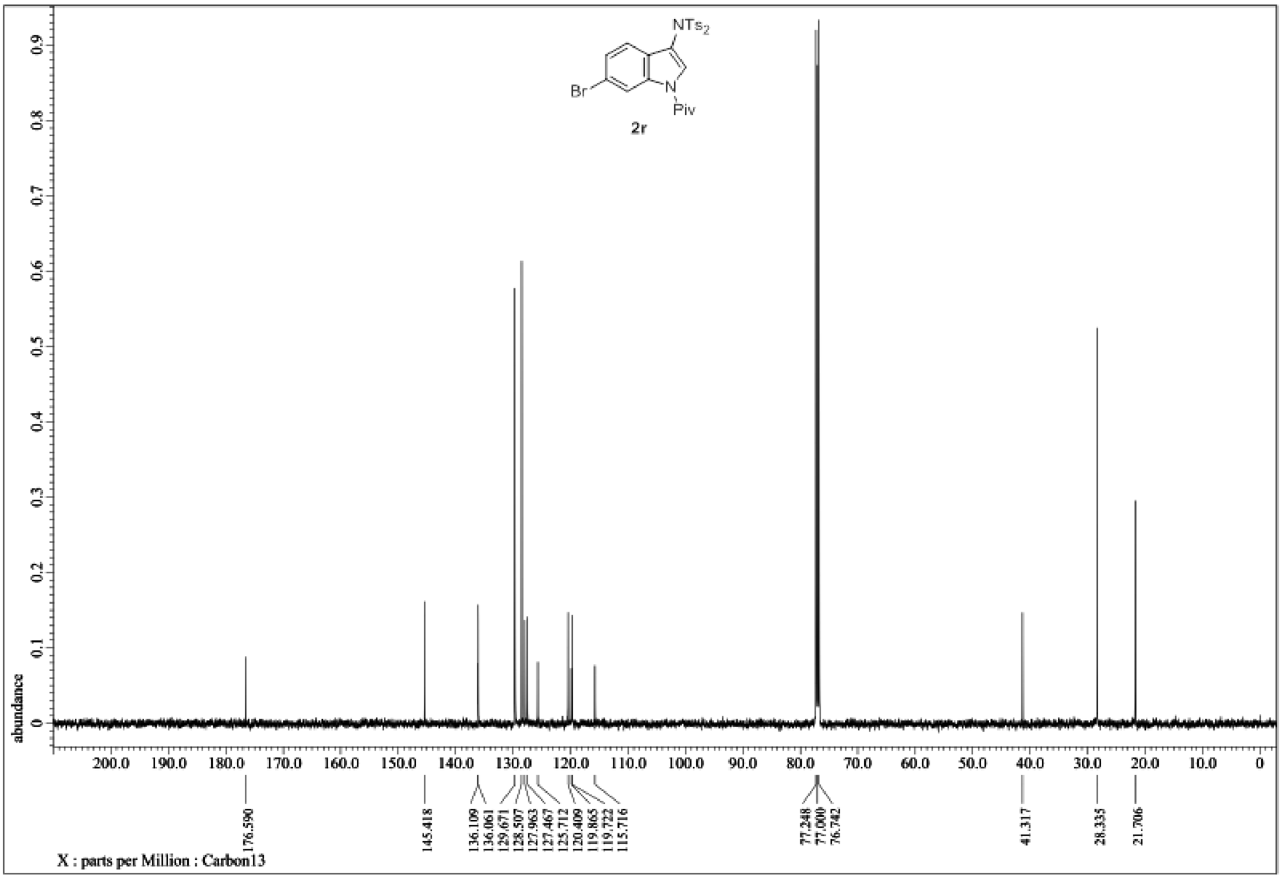
NTs2
Br
N
Piv
2r
abundance
X : parts per Million : Carbon13
176.590
145.418
136.109
136.061
129.671
128.507
127.963
127.467
125.712
120.409
119.865
119.722
115.716
77.248
77.000
76.742
41.317
28.335
21.706

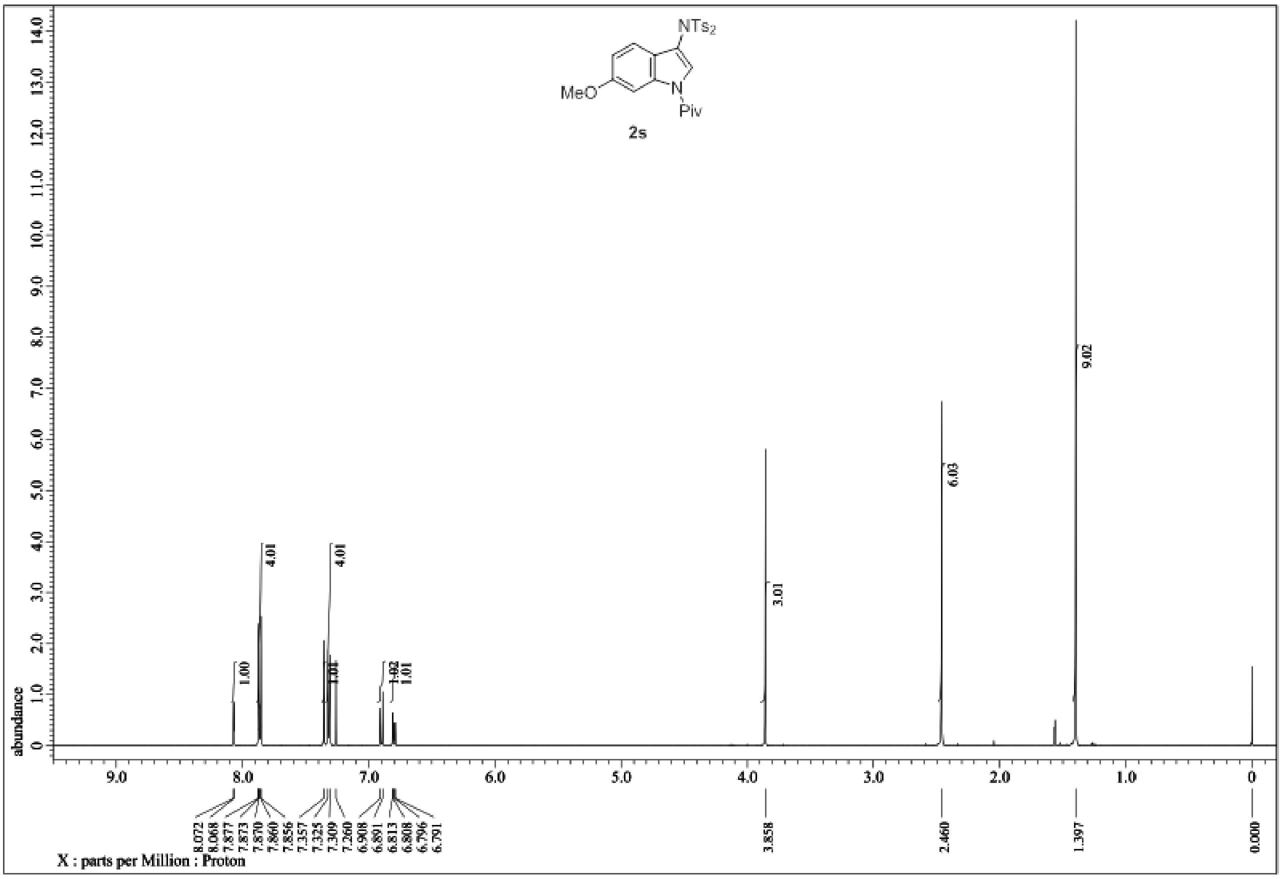
NTs2
MeO
N
Piv
2s
abundance
X : parts per Million : Proton

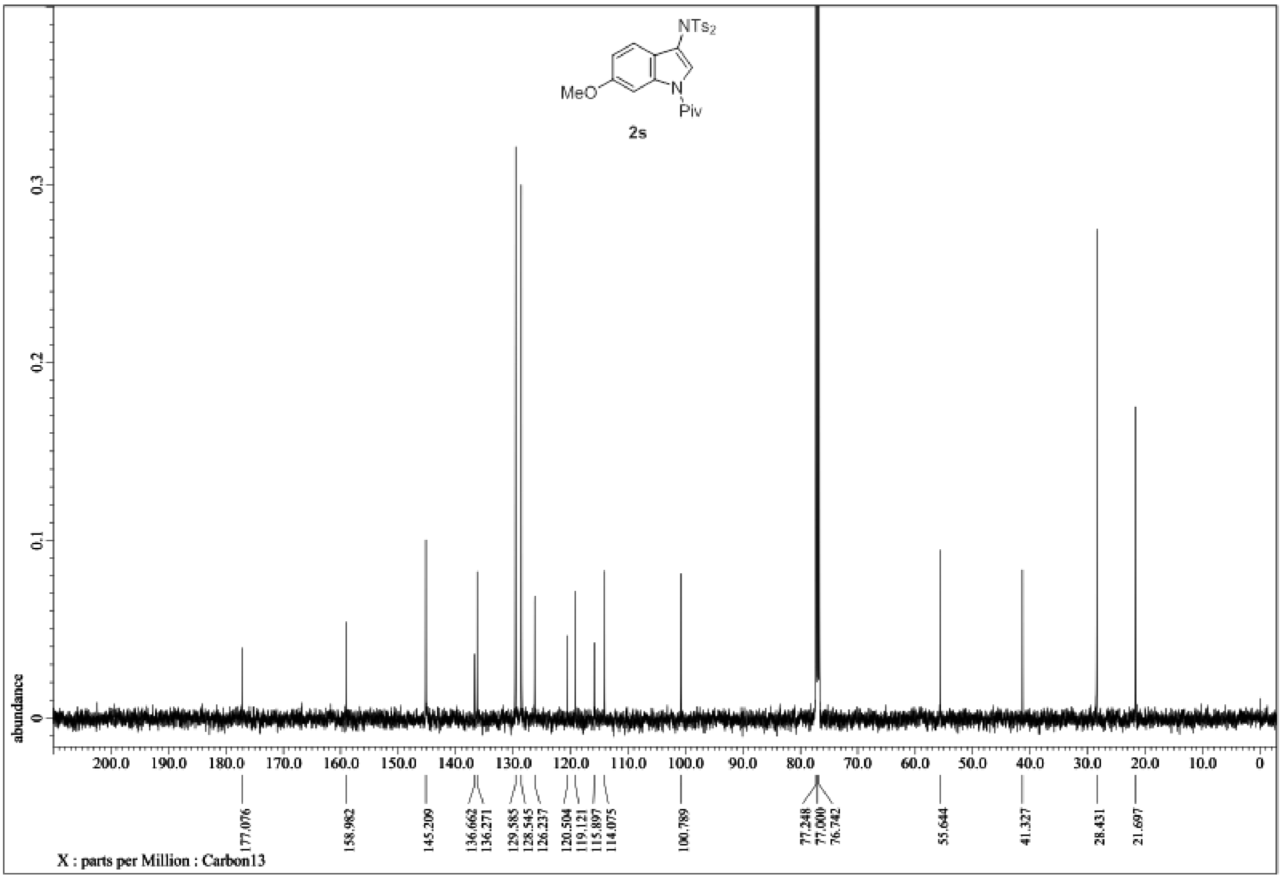
NTs2
MeO
N
Piv
2s
abundance
X : parts per Million : Carbon13

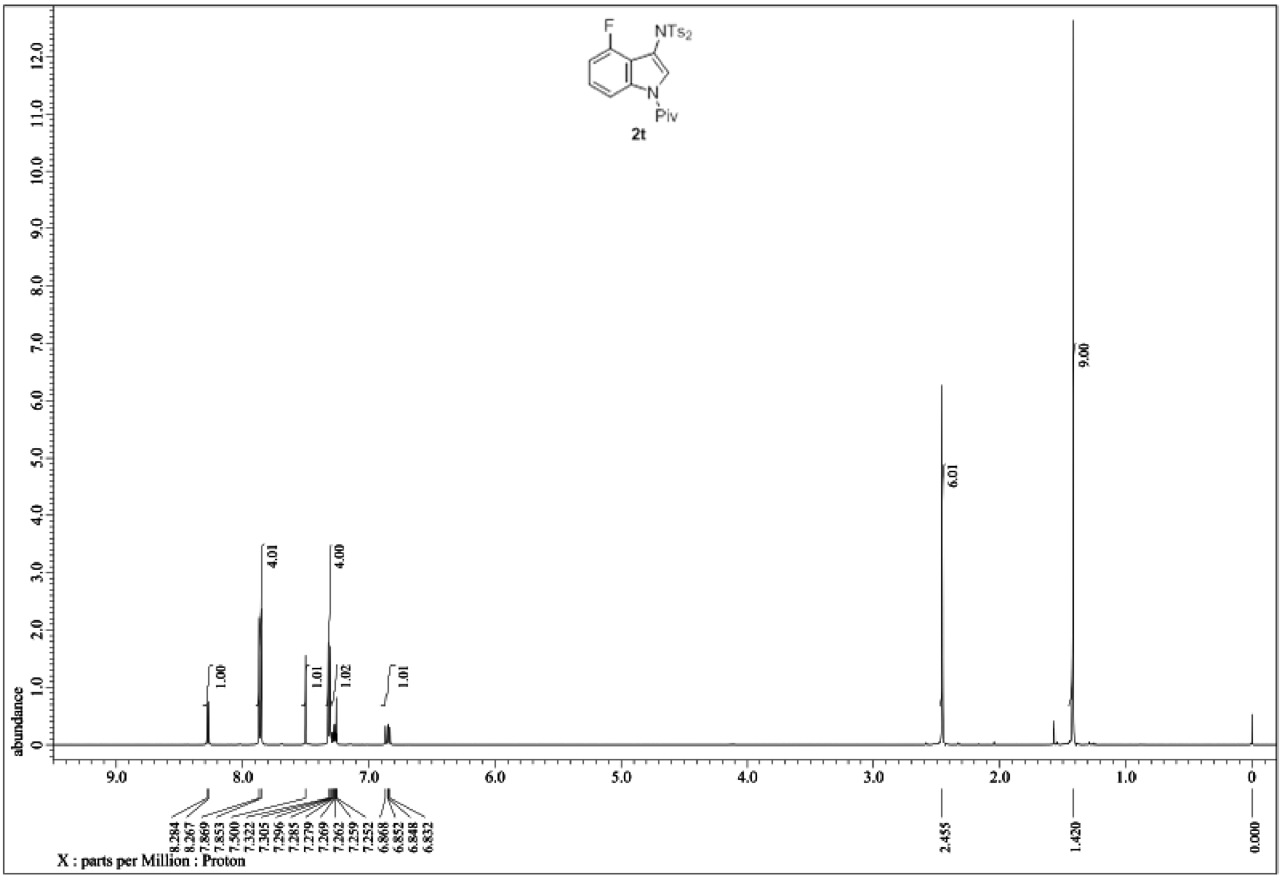
F
NTs2
N
Piv
2t
abundance
1.00
4.01
1.01
4.00
1.02
1.01
6.01
9.00
8.284 8.267 7.869 7.853 7.500 7.322 7.305 7.296 7.285 7.279 7.269 7.262 7.259 7.252 6.868 6.852 6.848 6.832
2.455
1.420
0.000
X : parts per Million : Proton

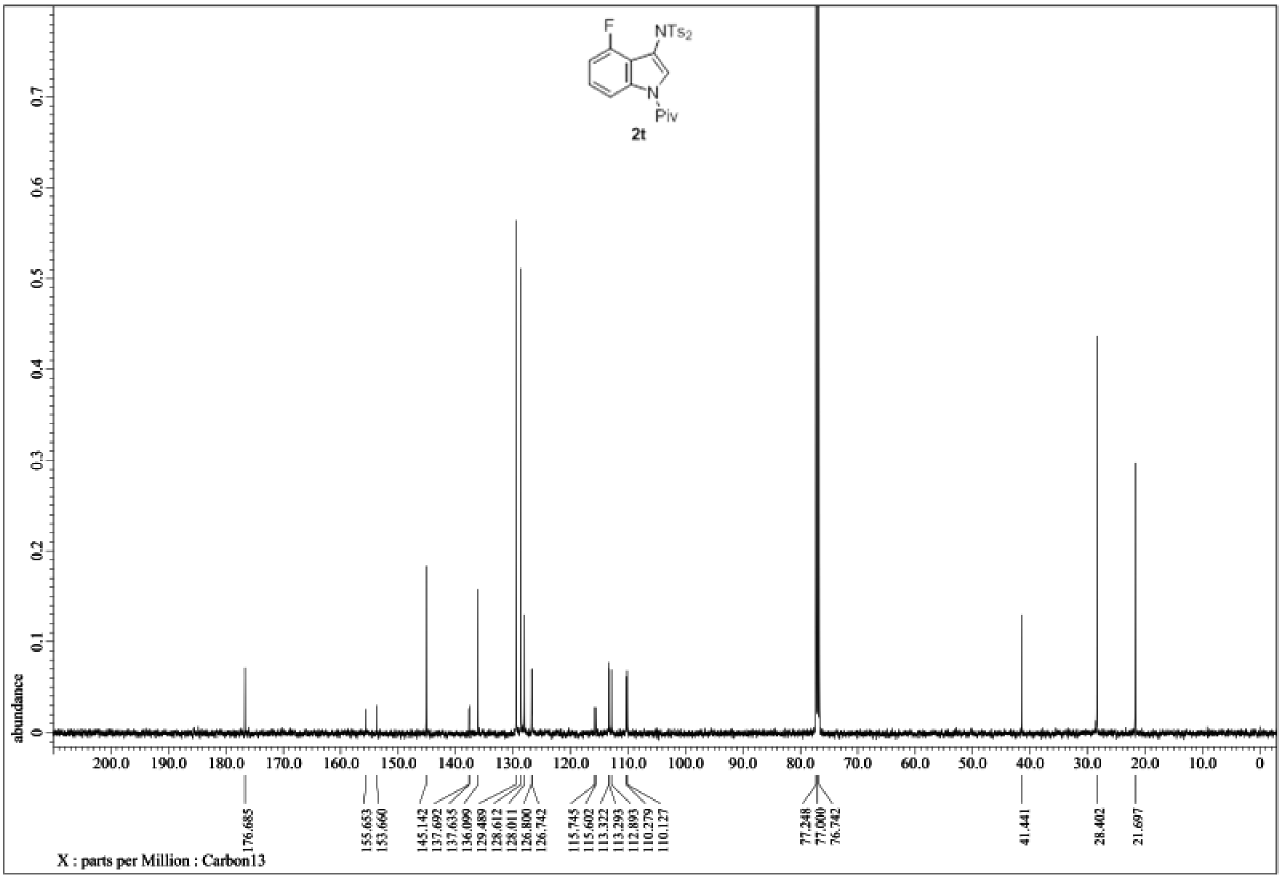
F
NTs2
N
Piv
2t
abundance
176.685
155.653 153.660
145.142 137.692 137.635 136.099 129.489 128.612 128.011 126.800 126.742
115.745 115.602 113.322 113.293 112.893 110.279 110.127
77.248 77.000 76.742
41.441
28.402
21.697
X : parts per Million : Carbon13

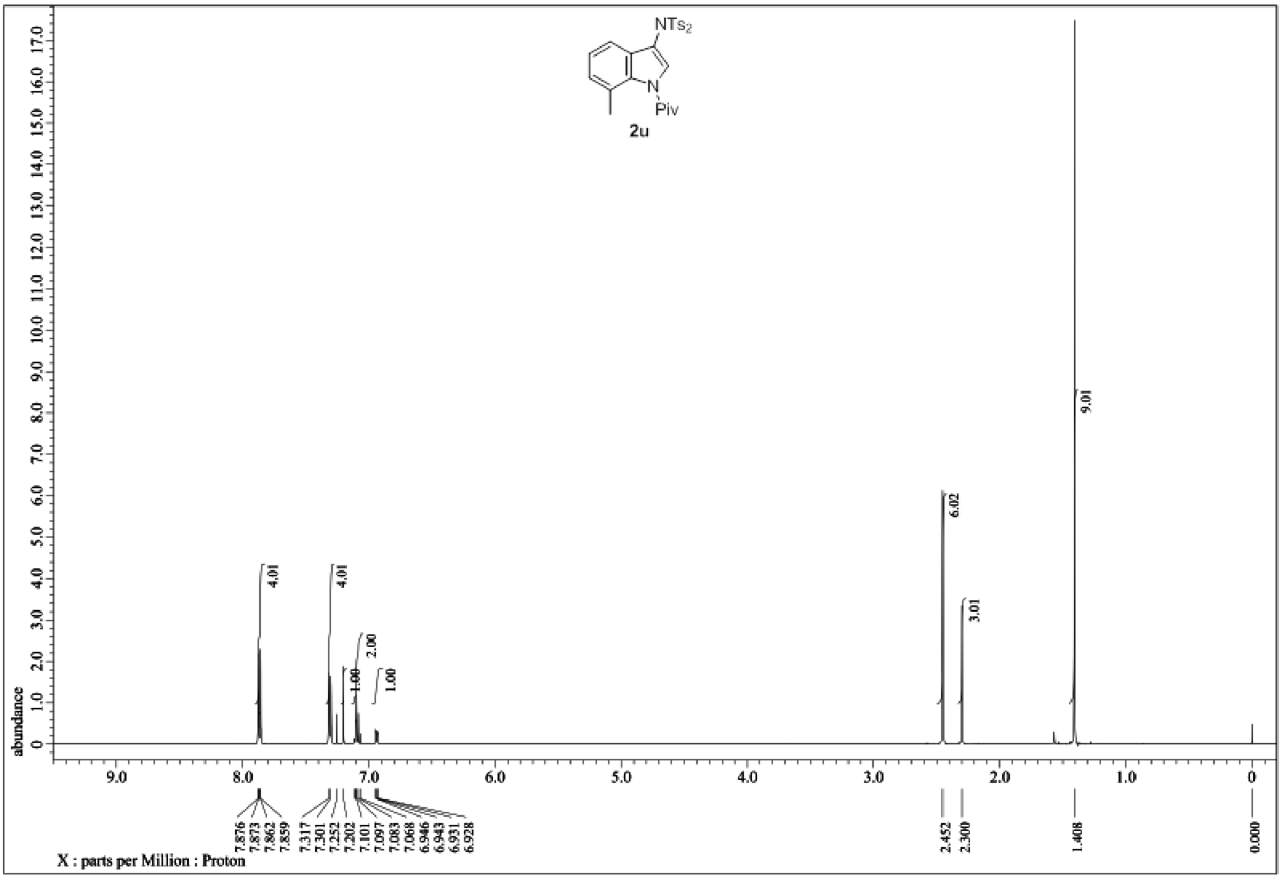
NTs2
N
Piv
2u
abundance
X : parts per Million : Proton
4.01
4.01
2.00
1.00
1.00
6.02
3.01
9.01
7.876
7.873
7.862
7.859
7.317
7.301
7.252
7.202
7.101
7.097
7.083
7.068
6.946
6.943
6.931
6.928
2.452
2.300
1.408
0.000

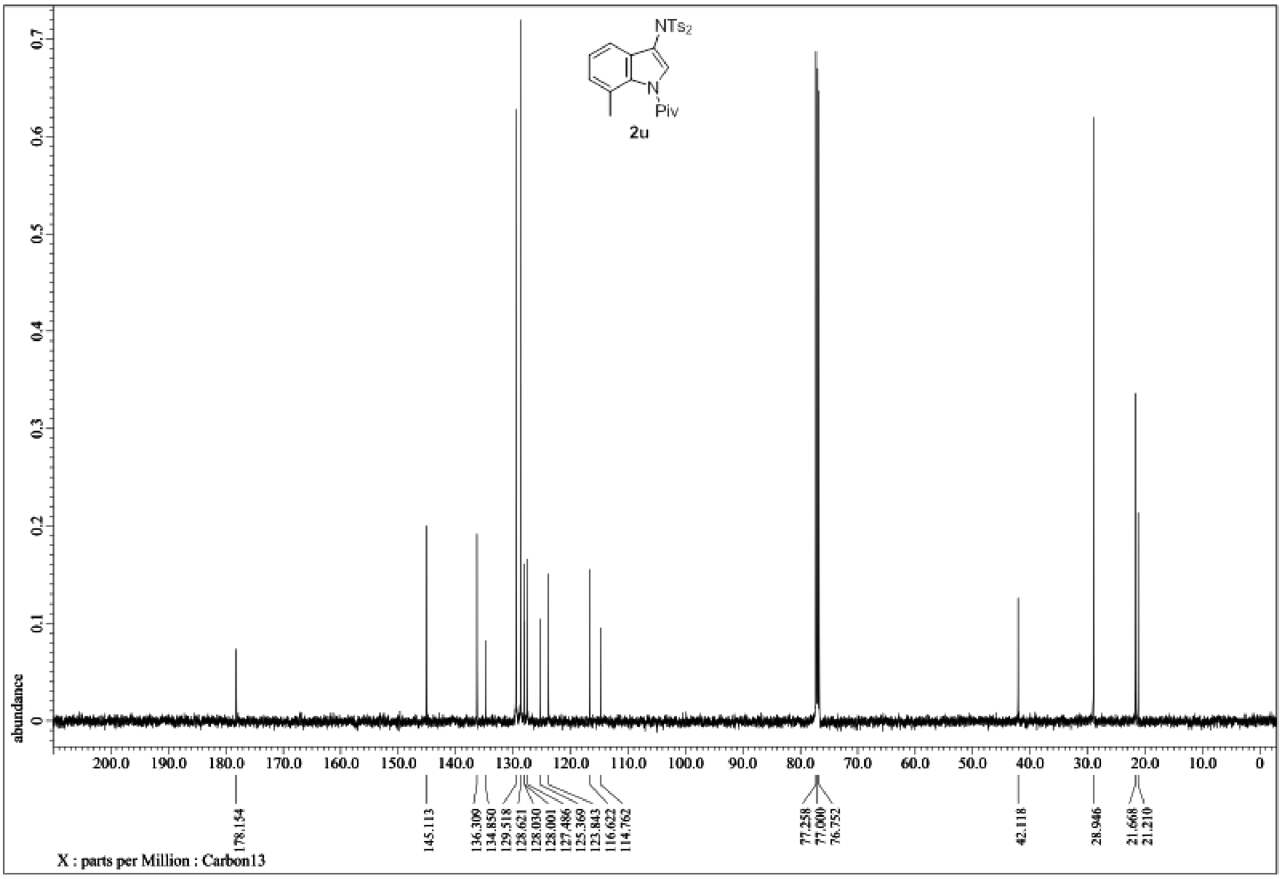
NTs2
N
Piv
2u
abundance
X : parts per Million : Carbon13
178.154
145.113
136.309
134.850
129.518
128.621
128.030
128.001
127.486
125.369
123.843
116.622
114.762
77.258
77.000
76.752
42.118
28.946
21.668
21.210

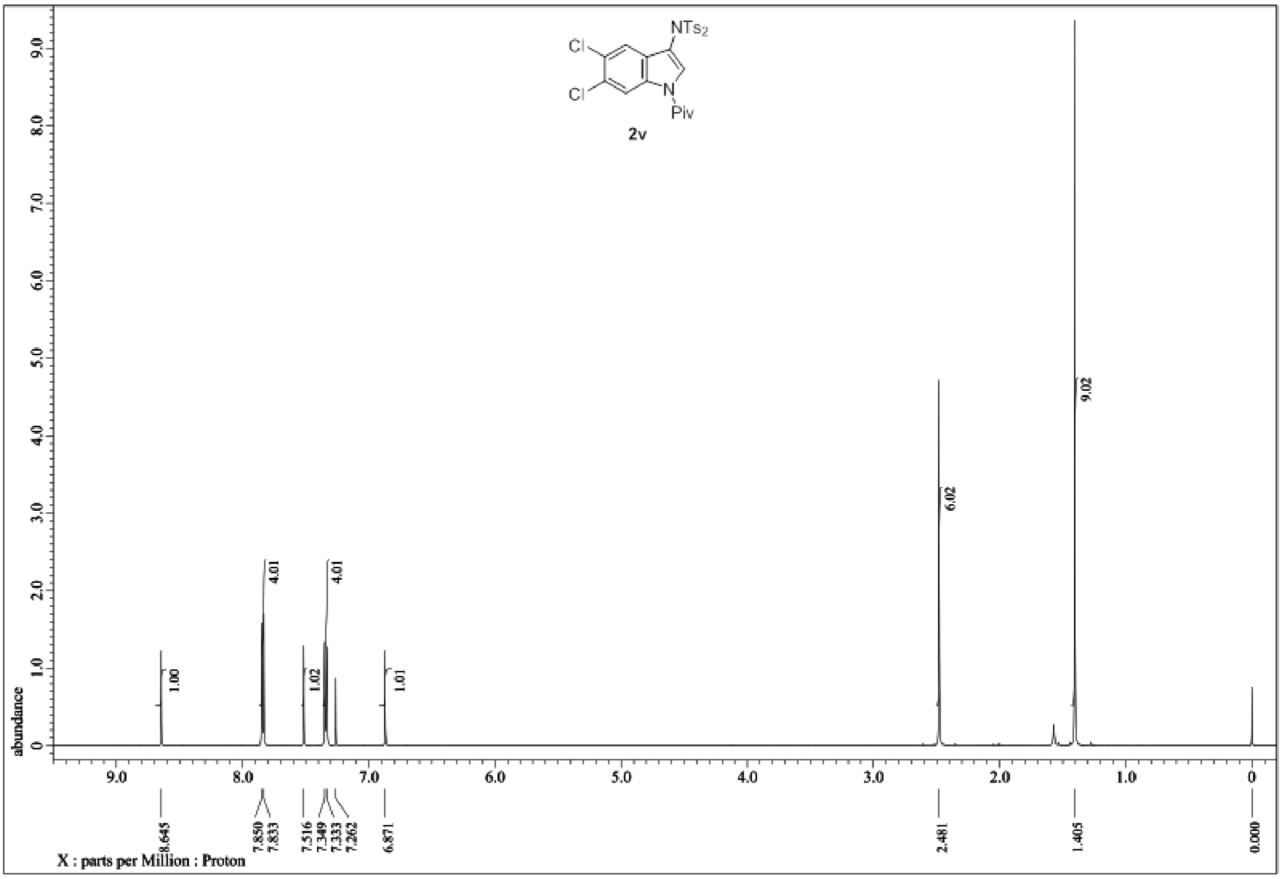
NTs2
Cl
Cl
N
Piv
2v
abundance
X : parts per Million : Proton
8.645
7.850
7.833
7.516
7.349
7.333
7.262
6.871
2.481
1.405
0.000
1.00
4.01
1.02
4.01
1.01
6.02
9.02

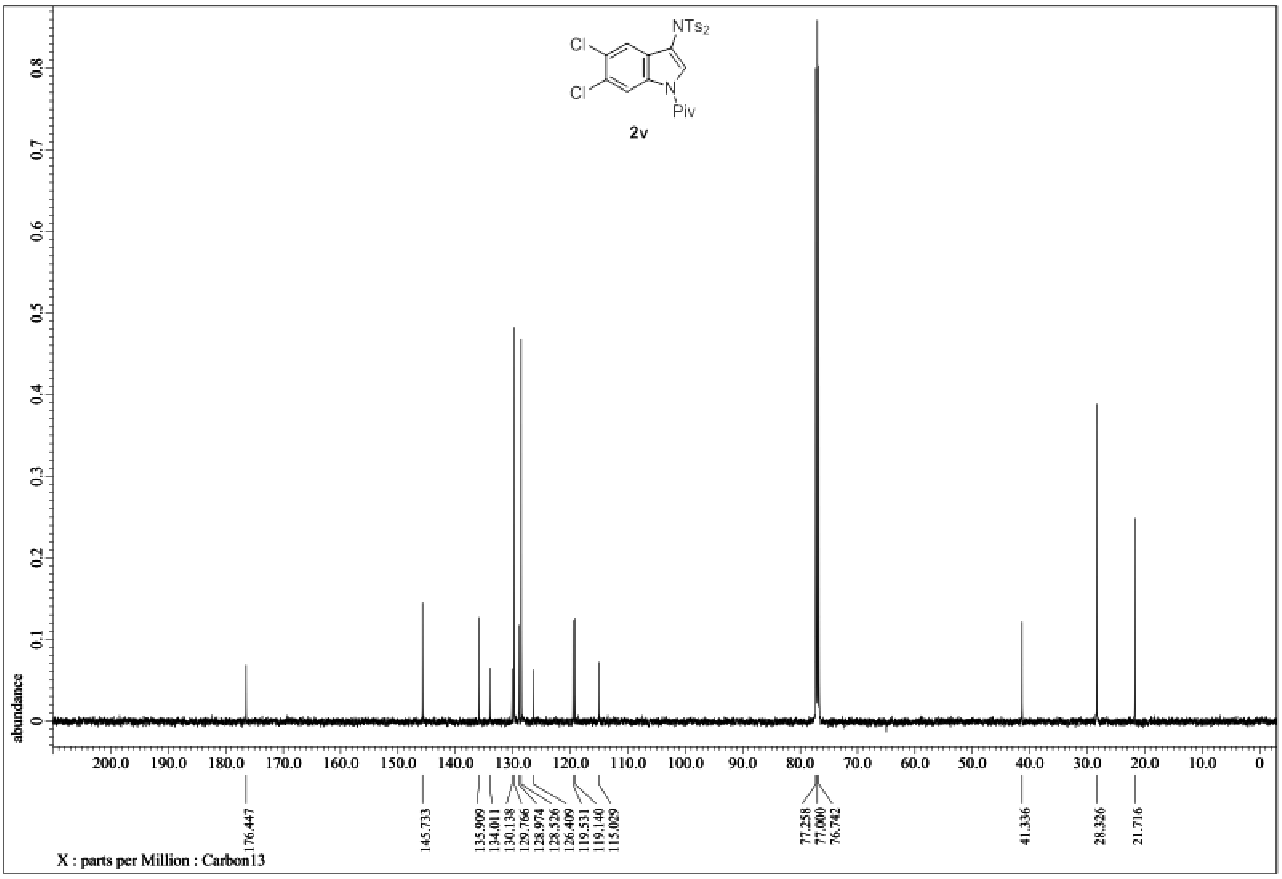
NTs2
Cl
Cl
N
Piv
2v
abundance
X : parts per Million : Carbon13
176.447
145.733
135.909
134.011
130.138
129.766
128.974
128.526
126.409
119.531
119.140
115.029
77.258
77.000
76.742
41.336
28.326
21.716

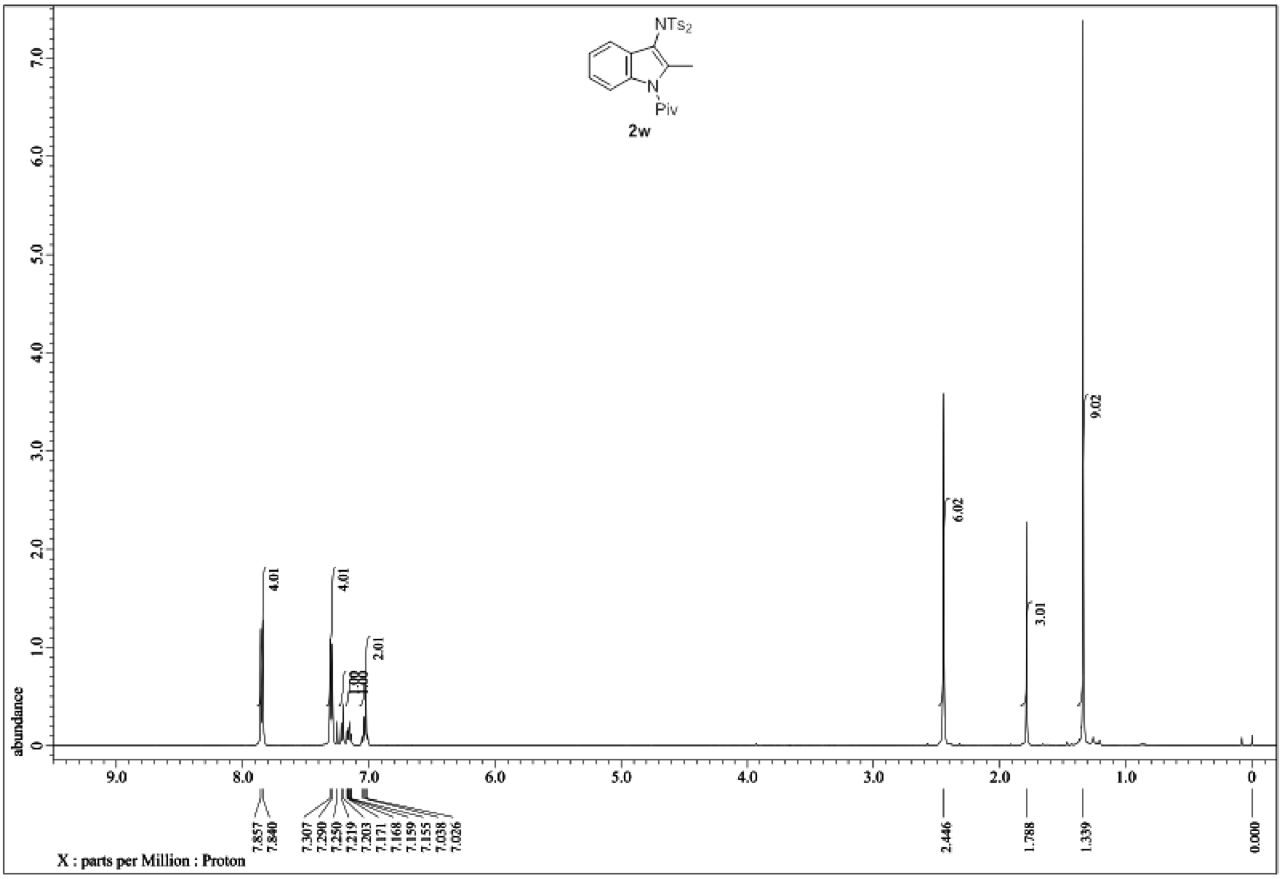
NTs2
N
Piv
2w
abundance
X : parts per Million : Proton
4.01
4.01
2.01
6.02
3.01
9.02
7.857
7.840
7.307
7.290
7.250
7.219
7.203
7.171
7.168
7.159
7.155
7.038
7.026
2.446
1.788
1.339
0.000

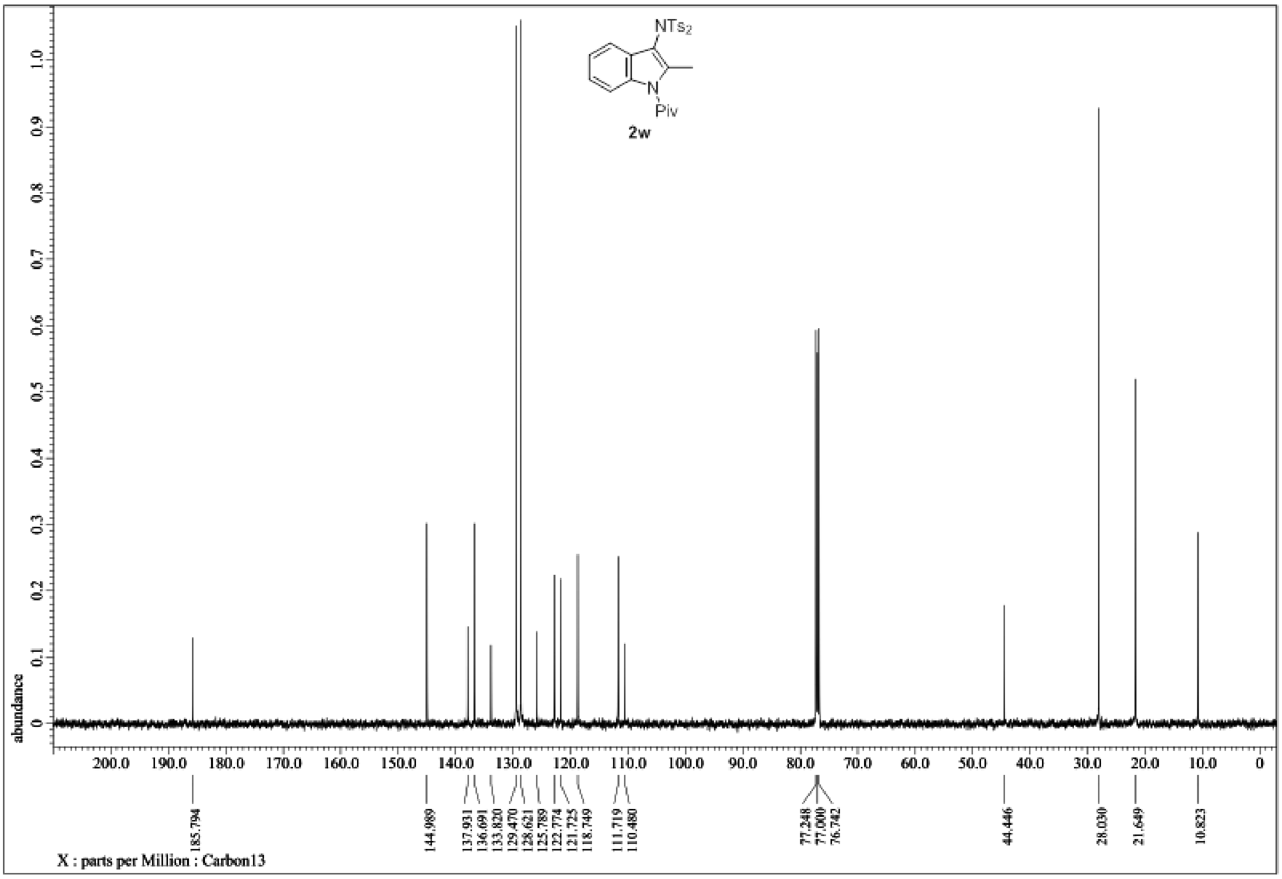
NTs2
N
Piv
2w
abundance
X : parts per Million : Carbon13
185.794
144.989
137.931
136.691
133.820
129.470
128.621
125.789
122.774
121.725
118.749
111.719
110.480
77.248
77.000
76.742
44.446
28.030
21.649
10.823

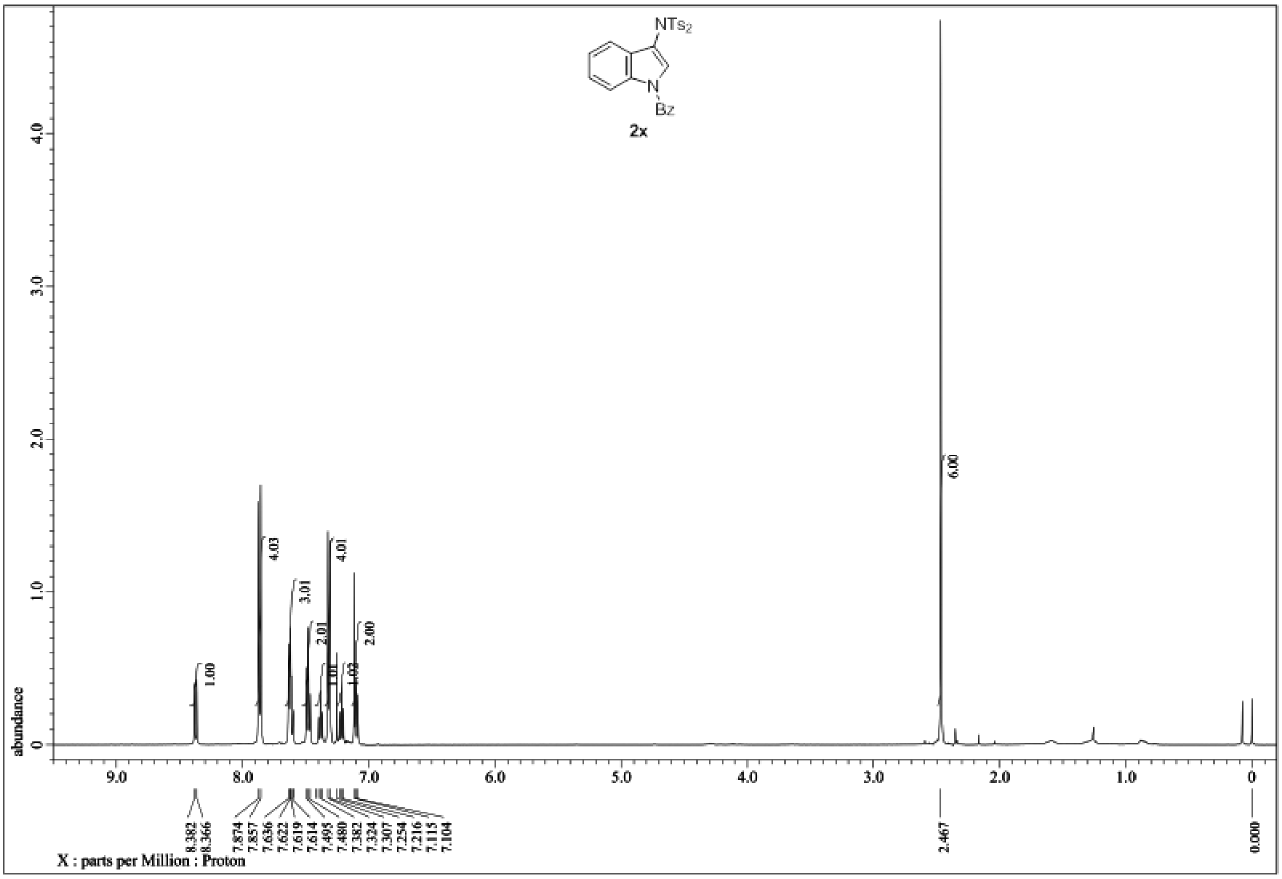
NTs2
N
Bz
2x
abundance
X : parts per Million : Proton
1.00
4.03
3.01
2.01
1.01
4.01
1.02
2.00
6.00
8.382
8.366
7.874
7.857
7.636
7.622
7.619
7.614
7.495
7.480
7.382
7.324
7.307
7.254
7.216
7.115
7.104
2.467
0.000

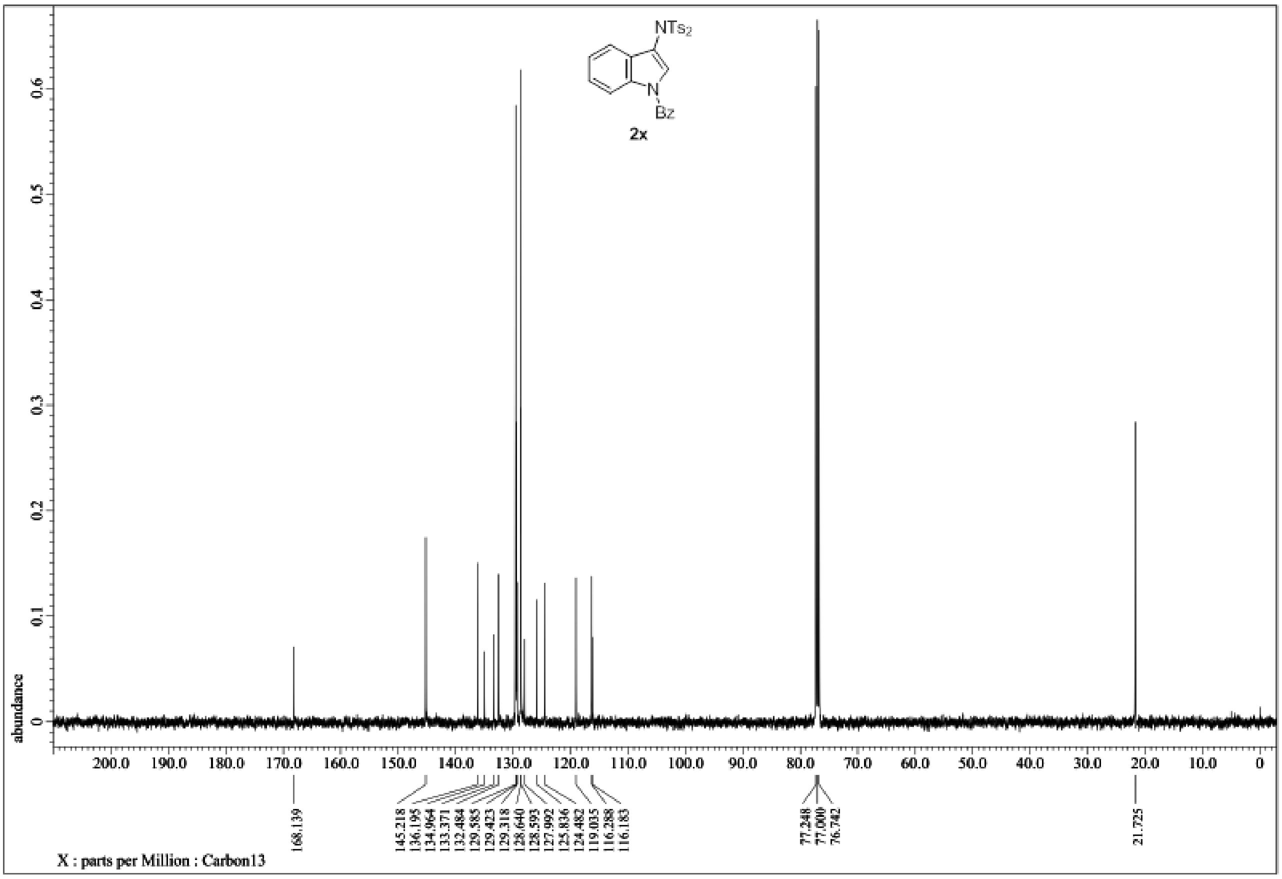
NTs2
N
Bz
2x
abundance
X : parts per Million : Carbon13
168.139
145.218
136.195
134.964
133.371
132.484
129.585
129.423
129.318
128.640
128.593
127.992
125.836
124.482
119.035
116.288
116.183
77.248
77.000
76.742
21.725

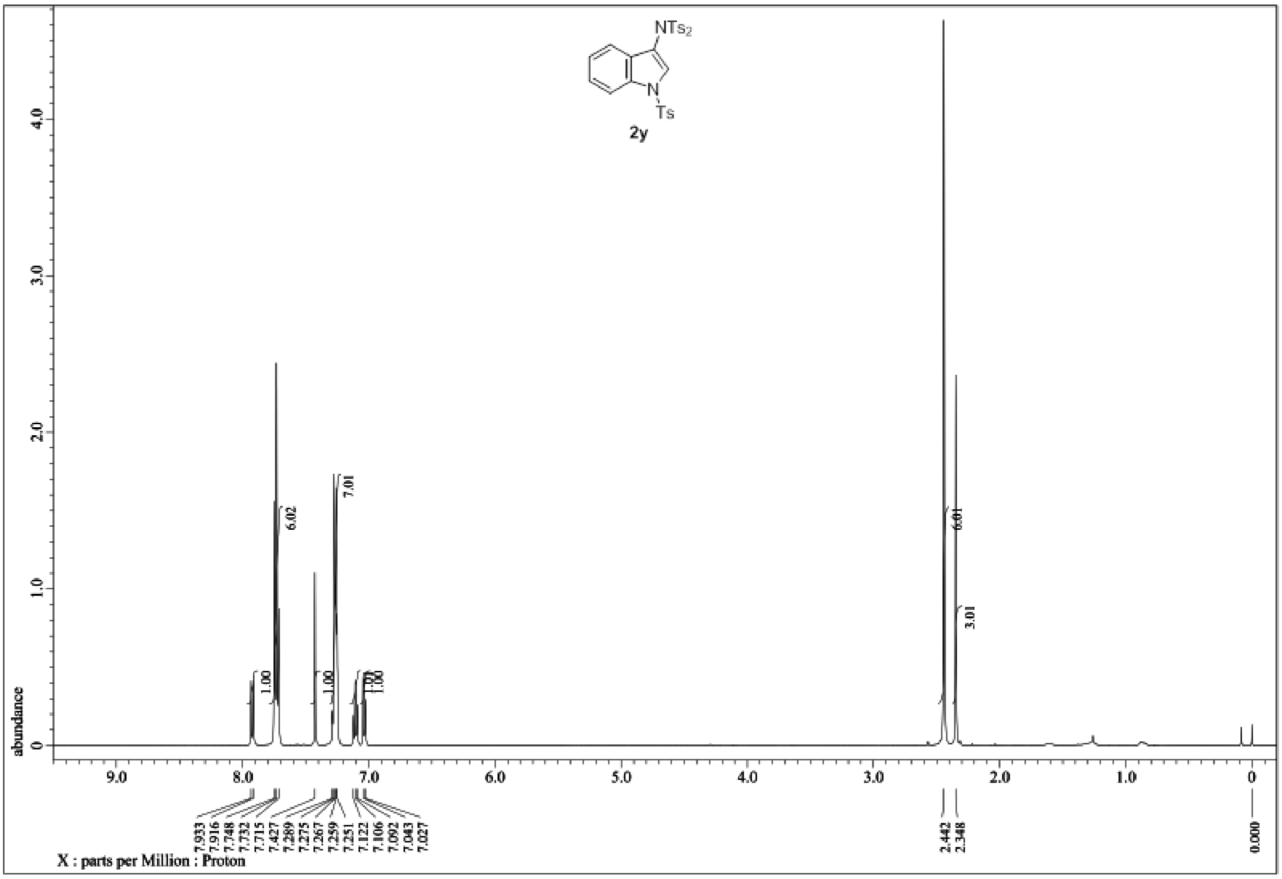
NTs2
N
Ts
2y
abundance
X : parts per Million : Proton
7.933
7.916
7.748
7.732
7.715
7.427
7.289
7.275
7.267
7.259
7.251
7.122
7.106
7.092
7.043
7.027
2.442
2.348
0.000
1.00
6.02
1.00
7.01
6.01
3.01

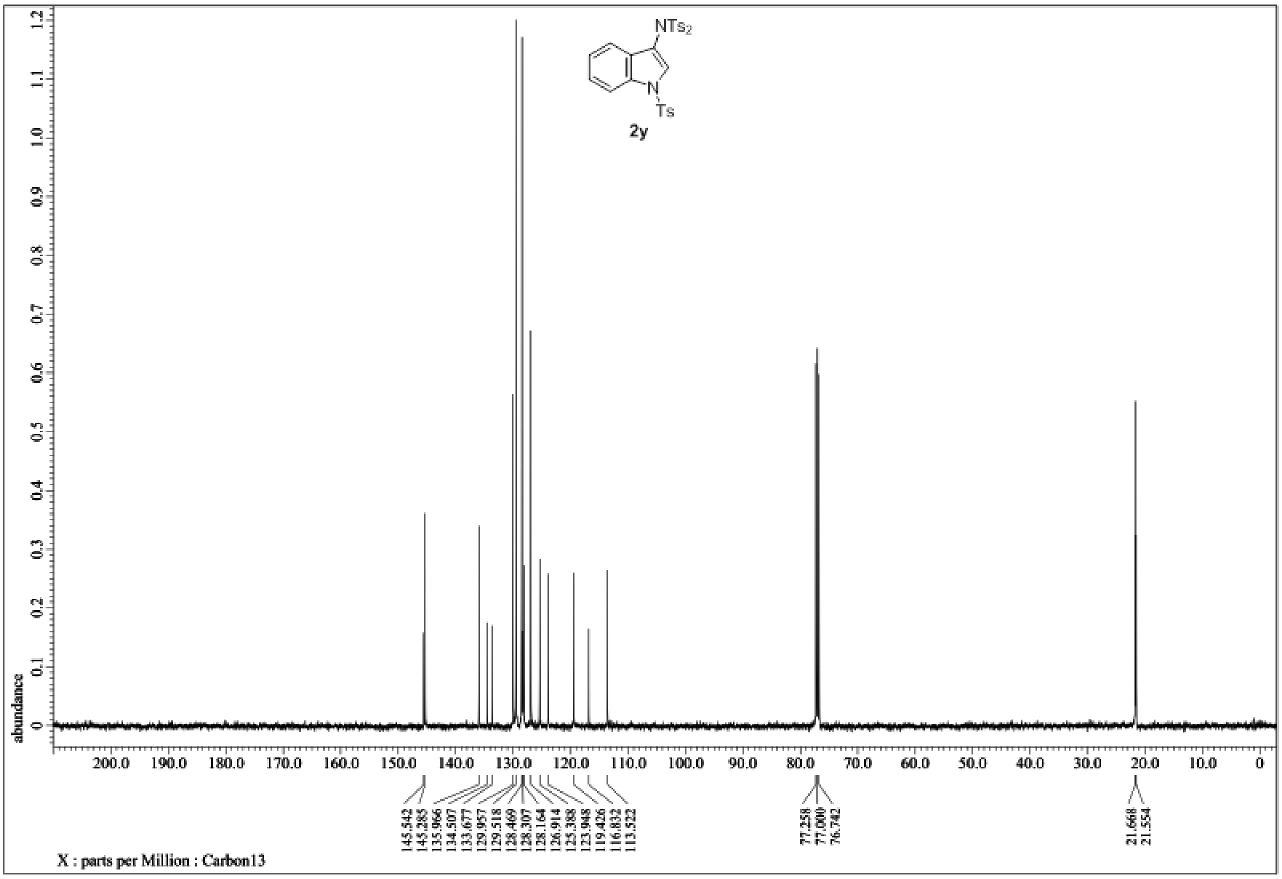
NTs2
N
Ts
2y
abundance
X : parts per Million : Carbon13
145.542
145.285
135.966
134.507
133.677
129.957
129.518
128.469
128.307
128.164
126.914
125.388
123.948
119.426
116.832
113.522
77.258
77.000
76.742
21.668
21.554
